# An Evaluation of the Implementation of Maternal Obesity Pathways of Care: A Mixed Methods Study with Data Integration

**DOI:** 10.1371/journal.pone.0127122

**Published:** 2015-05-27

**Authors:** Nicola Heslehurst, Sarah Dinsdale, Gillian Sedgewick, Helen Simpson, Seema Sen, Carolyn Dawn Summerbell, Judith Rankin

**Affiliations:** 1 Institute of Health & Society, Newcastle University, Newcastle upon Tyne, Tyne and Wear, United Kingdom; 2 Health and Social Care Institute, Teesside University, Middlesbrough, Teesside, United Kingdom; 3 Women and Children Centre, South Tees Hospitals NHS Foundation Trust, Middlesbrough, Teesside, United Kingdom; 4 School of Medicine, Pharmacy & Health, Durham University, Stockton on Tees, Teesside, United Kingdom; Iran University of Medical Sciences, Islamic Republic of Iran

## Abstract

**Objectives:**

Maternal obesity has multiple associated risks and requires substantial intervention. This research evaluated the implementation of maternal obesity care pathways from multiple stakeholder perspectives.

**Study Design:**

A simultaneous mixed methods model with data integration was used. Three component studies were given equal priority. 1: Semi-structured qualitative interviews explored obese pregnant women’s experiences of being on the pathways. 2: A quantitative and qualitative postal survey explored healthcare professionals’ experiences of delivering the pathways. 3: A case note audit quantitatively assessed pathway compliance. Data were integrated using following a thread and convergence coding matrix methods to search for agreement and disagreement between studies.

**Results:**

Study 1: Four themes were identified: women’s overall (positive and negative) views of the pathways; knowledge and understanding of the pathways; views on clinical and weight management advice and support; and views on the information leaflet. Key results included positive views of receiving additional clinical care, negative experiences of risk communication, and weight management support was considered a priority. Study 2: Healthcare professionals felt the pathways were worthwhile, facilitated good practice, and increased confidence. Training was consistently identified as being required. Healthcare professionals predominantly focussed on women’s response to sensitive obesity communication. Study 3: There was good compliance with antenatal clinical interventions. However, there was poor compliance with public health and postnatal interventions. There were some strong areas of agreement between component studies which can inform future development of the pathways. However, disagreement between studies included a lack of shared priorities between healthcare professionals and women, different perspectives on communication issues, and different perspectives on women’s prioritisation of weight management.

**Conclusion:**

The differences between healthcare professionals’ and women’s priorities and perspectives are important factors to consider when developing care pathways. Shared perspectives could help facilitate more effective implementation of the pathway interventions that have poor compliance.

## Introduction

There is a strong international evidence-base which shows an association between increased pre-pregnancy body mass index (BMI) and adverse pregnancy outcomes for both the mother and her child. Women with a BMI >30kg/m^2^ (clinically defined as obese) before pregnancy or in early pregnancy have a significantly increased risk of mortality, and comorbidities such as gestational diabetes and pre-eclampsia[1–3]. Outcomes for the child also include reduced breastfeeding rates, increased risk of congenital anomaly, and neonatal mortality[4–6]. Pregnancy is also a critical period for women’s development of long-term health conditions such as obesity and type 2 diabetes[7,8]. There is increasing evidence that the fetal environment is associated with the development of obesity and related disease among the offspring of obese women[9,10]. Managing complex pregnancies has resource implications for health services, such as longer hospital stay, increased caesarean section rates, treating maternal infections, and neonatal intensive care[11,12]. These additional interventions are associated with increased costs for health services compared with managing pregnancies of women within the recommended BMI range[12–14].

A number of countries have produced clinical guidelines for maternal obesity and gestational weight gain in recent years[15–20]. In the UK, maternal obesity is considered to be an independent risk factor which requires more intensive antenatal intervention (e.g. screening for gestational diabetes, anaesthetic assessments, and obstetrician led care)[21]. In England, the number of women requiring this level of antenatal care due to obesity has more than doubled over two decades[22]. Similar trends in maternal obesity have been identified throughout the rest of the UK[23,24] and internationally[25–29]. In the UK, 2010 marked a heightened policy focus on maternal obesity with the publication of two sets of national guidelines specific to the clinical management of maternal obesity[21], and weight management during pregnancy[30]. The National Health Service Litigation Authority (NHSLA) has also published standards of care for maternal obesity which maternity services must adhere to in order to achieve accreditation from the Clinical Negligence Scheme for Trusts (CNST)[31,32].

Despite the international publication of guidelines and standards of care, the existing evidence-base identifies multiple barriers to healthcare professionals’ maternal obesity and weight management practice[33]. A multidisciplinary steering group in a large NHS Trust in the Northeast of England aimed to overcome barriers to practice by developing care pathways for the management of maternal obesity. The group consisted of healthcare professionals (including midwives, obstetricians, dietitians, anaesthetists, diabetes clinicians and nurse specialists), and academic and primary care partners (including representatives from specialist weight management services, health improvement services and commissioners). Three maternity care pathways were developed for the clinical and weight management of maternal obesity, defined by the BMI at first antenatal contact (see S1 Table). The care pathways incorporated antenatal, intrapartum, and postnatal care. The pathways’ content were developed using the best available published evidence, including national guidelines and research evidence, and went through an iterative process of being updated in line with developments in national recommendations and evidence[3,21,23,30,34]. When there was an absence of appropriate evidence, the content was developed based on discussion and agreement of expertise among the multi-disciplinary steering group. A pathway profoma was developed to be filed in women’s handheld antenatal records, with sections to be completed by a healthcare professional at specific contacts. Two leaflets were developed to be used alongside the pathways: a patient information leaflet on maternal obesity, and a leaflet to aid healthcare professionals maternal obesity practice. Prior to implementation, the pathways were reviewed and given favourable feedback from the NHS Trust Maternity Services Liaison Committee, which includes service user representatives.

Evaluating the implementation of a new model of care such as these obesity pathways can aid our understanding of implementation successes and failures. Multiple perspectives can be explored using a mixed methods approach to evaluation. Mixed methods research has two predominant models: the sequential model (or combination of methods), and the simultaneous model (or integration of methods). Sequential models involve carrying out component studies to inform subsequent phases, and the component studies are usually prioritised (e.g. a qual→QUANT model prioritises the quantitative component, which has been informed by the qualitative component). Simultaneous models involve component studies being carried out concurrently usually with equal priority, and data are integrated during analysis (e.g. a QUAL+QUANT model). The simultaneous model is considered to be “true” mixed methods research, especially when data are integrated from multiple methods using a triangulation approach to search for convergent or dissonant findings. However, published mixed methods research often describes the sequential model, lacks the integration aspect of the simultaneous model, or lacks a description of the integration process[35–39]. Triangulation protocols for simultaneous models recommend that the data collection and analyses of component studies should be carried out and reported separately, with integration at the stage of interpretation[35,37,40].

This mixed methods study aimed to evaluate the implementation of the care pathways from multiple perspectives. The objectives were:

- To explore obese pregnant women’s understanding and experiences of being on the care pathways, and their views on the information and support received
- To explore healthcare professionals’ awareness and experiences of delivering the pathways, and any perceived training needs
- To assess compliance with the pathways to determine which aspects had been implemented to an acceptable level
- To identify how the multiple perspectives related to one another through integration of data

## Methods

**Fig 1:**
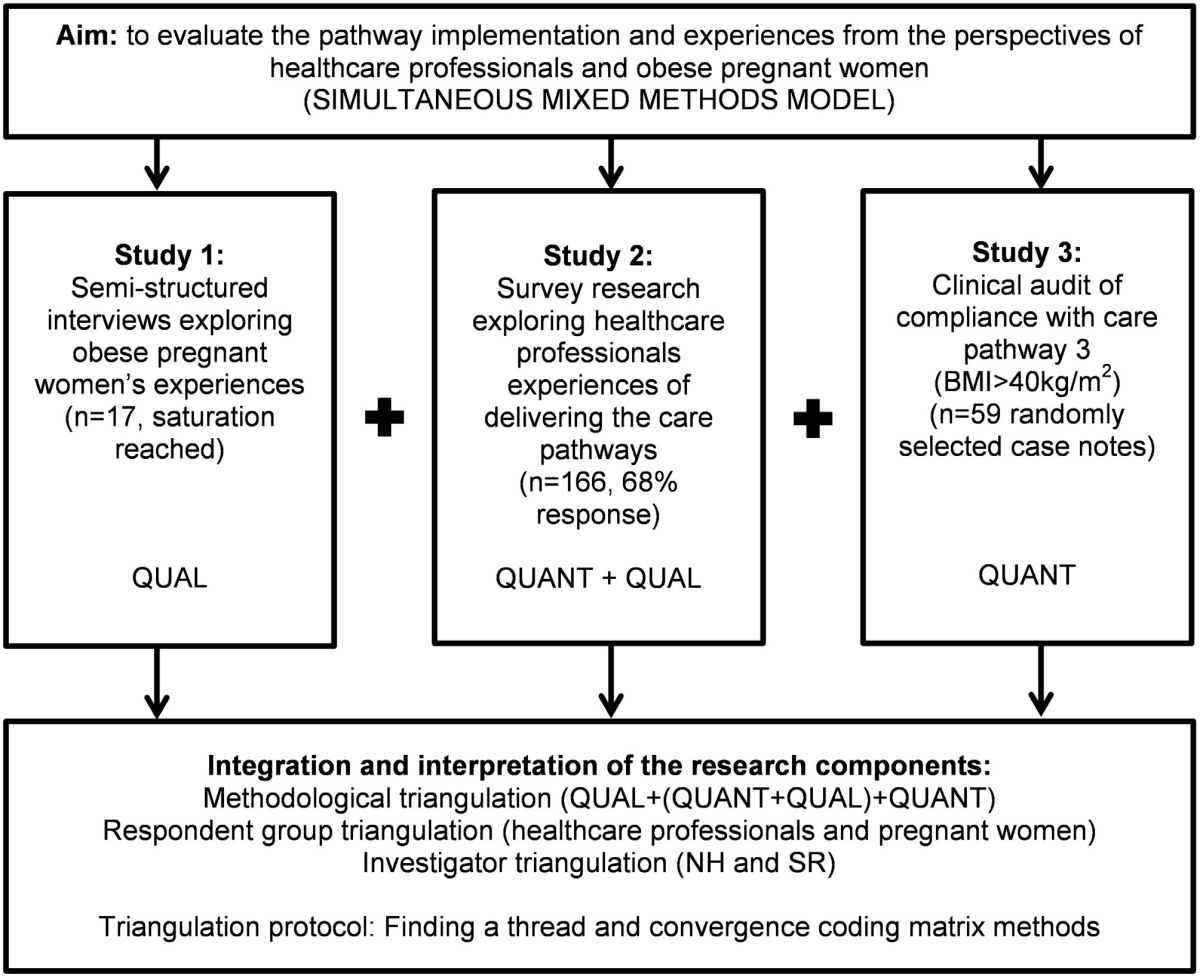
Flowchart showing simultaneous mixed methods model and multiple triangulation techniques. Fig 1 shows the mixed methods model including the research aim; studies 1, 2 and 3; the integration of data; and the triangulation of methods, respondent group and investigators.

A simultaneous mixed methods model was used in this study, comprising of three component studies with equal priority and data integration during interpretation. Multiple triangulation techniques were employed: methodological triangulation, data triangulation, and investigator triangulation. Methodological triangulation incorporated qualitative and quantitative research. Data were triangulated from multiple sources including healthcare professionals, obese pregnant women, and antenatal records. Two investigators (NH and SD) triangulated their independent data analysis of the component studies, and both were involved in the integration and interpretation (Fig 1). Following the recommendations made in triangulation protocols[35,37], this paper reports each individual component study separately followed by data integration.

Research Ethics: Study 1 involved research with NHS patients and therefore NHS ethical approval was sought and gained from Newcastle & North Tyneside 2 research ethics committee, as well as from Teesside University School of Health and Social Care ethics committee. Written informed consent was obtained from the participants in person for study 1. Study 2 involved questionnaire research with healthcare professionals and was therefore exempt from NHS ethical approval procedures; however, approval was granted by Teesside University School of Health and Social Care ethics committee. The questionnaires included information about the study and completion was regarded as informed consent. South Tees NHS Trust research and development committee approved studies 1 and 2. In line with the General Medical Council guidelines for confidentiality and consent for local clinical audit[41], study 3 was carried out by medical staff within the NHS trust who were members of the maternity care team. Patients in the NHS Trust are advised of the use of their data for clinical audit in a patient information leaflet, and of their right to object and opt out. As study 3 was a clinical audit, it was exempt from NHS and university ethical approval procedures.

### Study 1 methods: semi-structured interviews exploring obese pregnant women’s experiences of the care pathways.

One-to-one semi-structured interviews were carried out with obese pregnant women (BMI>30kg/m^2^) to explore their understanding and experiences of the care pathways, and their views on the information and support provided. A convenience sampling strategy was used, and women were recruited during routine antenatal contacts by a member of the research team (NH). The antenatal contacts utilised for recruitment were oral glucose tolerance test (OGTT) clinics, and a healthy lifestyle clinic for women with a BMI >40kg/m^2^. The antenatal healthy lifestyle clinic involved an initial consultation with a public health consultant midwife which was additional to routine antenatal care. This consultation was followed by a one-to-one consultation with a dietitian. Women referred to the healthy lifestyle clinic could continue to attend throughout their pregnancy, and would have the opportunity to see both the consultant midwife and dietitian at each visit.

Following written informed consent, semi-structured interviews (see S2 Table) were audio-recorded, transcribed verbatim and analysed by two researchers independently (NH and SD) using a framework analysis approach. Framework analysis was developed for research with pre-defined objectives[42], and offers a systematic and comprehensive approach which is based on the fundamental accounts of participants[43]. Potential limitations of the approach relate to its deductive nature which leads to the potential forcing of data into *a priori* themes rather than formulating data derived themes[43]. Nevertheless, an element of flexibility and dynamicity exists as thematic frameworks can be refined throughout the analysis to reflect the data[44]. Both researchers independently followed stages one to three of the analytical process described by Ritchie and Spencer[44] (familiarisation, identifying a thematic framework, and indexing), and triangulated their analyses during stages four and five (charting, and mapping and interpretation). Investigator triangulation aimed to reduce researcher bias and address the deductive limitation of framework analysis by ensuring data driven themes were incorporated. The final coding framework included both *a priori* themes and data driven themes.

Recruitment continued until no new themes emerged and saturation was apparent. The results are presented narratively with supporting quotations. Data that are underlined represent any emphasis that the participants placed on words; ellipses represent unrelated data removed from quotations; and square brackets are used when contextual information is added for clarity.

### Study 2 methods: survey research exploring healthcare professionals’ experiences of delivering the clinical pathways.

A postal survey was developed and distributed to all healthcare professionals responsible for delivering the obesity care pathways. Disciplines included hospital- and community-based midwives, consultant obstetricians, consultant anaesthetists, and trainee obstetricians (including specialist registrars and senior house officers). In the absence of an existing validated questionnaire, the questionnaire content was developed based on the objectives of the study and using existing evidence on barriers to clinical practice (largely qualitative evidence). The questionnaire was piloted with five healthcare professionals representing the specialities to be included in the survey. Four pilot participants provided feedback which confirmed the content did not need refining. However, the layout of the final questionnaire was adapted to be more user-friendly.

The questionnaire was designed and distributed in accordance with evidence-based survey recommendations[45–47]. These recommendations aim to reduce survey error by incorporating the methodological principles of social exchange theory, where the response of a participant is reflective of the perceived benefits and costs of participating[45]. Up to two follow up questionnaires were posted to non-responders with the aim of increasing the response rate and sample representativeness. A response rate of 70% was anticipated using this method[45].

The questionnaire included quantitative and qualitative questions (see S1 Questionnaire). For the quantitative questions, respondents were asked to indicate their level of agreement with statements using a five point scale (1: strongly agree to 5: strongly disagree). Descriptive analyses were carried out to explore the majority response for each question (mean and standard deviation (SD)), and pattern of response (percentage response for each level of agreement from 1 to 5). Independent t-tests (using the Levenes test for variance) determined any significant difference between midwifery and clinician participants. The qualitative components of the questionnaire included open ended questions with free text space for participants to elaborate on their perspectives or experiences. The qualitative questionnaire data were analysed using a framework which was developed to represent the topics included in the questionnaire, as well as incorporating data driven sub-themes. The framework analysis followed the same methods described in component study 1. The questionnaire qualitative analyses were triangulated with the associated quantitative analysis to add depth and interpretation.

### Study 3 methods: clinical audit of compliance with care pathway 3 (BMI>40kg/m^2^).

The clinical audit investigated compliance with care pathway 3 which was developed for pregnant women with the greatest weight-related risk, and includes the highest level of intervention among three care pathways (see S1 Table). Therefore the research team felt that assessing the compliance with this pathway was of greatest importance.

The clinical audit was carried out by the NHS Trust clinical audit team and medical staff. An audit pro-forma was developed to reflect the care pathway (by SS and NH in collaboration with the clinical audit team). Data were collected retrospectively using information from handheld antenatal records for randomly selected women who had been discharged from care pathway 3 (random selection was carried out by the clinical audit team, data collection by two trainee obstetricians supervised by SS a consultant obstetrician). Data were analysed descriptively using percentage compliance (by SS). Determining whether compliance was acceptable or unacceptable used a 75% cut off. This was based on the NHSLA accreditation definition which deems ≥75% compliance with standards of care to be acceptable, and <75% to be unacceptable compliance[32]. Compliance was assessed for individual pathway components, as well as overall compliance of antenatal, intrapartum and postnatal components of the pathway.

### Methods for integration of the research components

The triangulation protocol used the following methods for the integration of the individual studies: 1) following a thread, and 2) convergence coding matrix[35,37]. Two researchers carried out the integration and interpretation (NH and SD).

The following a thread method involved identifying a concept from study 1, and then searching studies 2 and 3 for related data (threads). This process continued until all concepts from study 1, and threads from studies 2 and 3 had been identified. Study 2 was then searched for any additional concepts, and any threads from study 3 were identified. Finally, study 3 was reviewed to identify if there were any additional concepts not identified in studies 1 and 2.

A convergence coding matrix was used to integrate threads into themes and meta-themes. This process involved actively searching and comparing the threads for any patterns (themes) that arose. Themes were then grouped based on similarity of concept and interpreted to identify the meaning of these themes (generating meta-themes). Finally, meta-themes were searched for agreement and disagreement between research studies. Agreement and disagreement was defined as[35,37]:

1. Convergence: where findings directly agree
2. Complementarity: findings offer complimentary information on the same issue
3. Dissonance: findings appear to contradict one another
4. Silence: themes arising from one component study but not others

## Results

Results are presented separately for studies 1–3, and the integration.

### Study 1 results: semi-structured interviews exploring obese pregnant women’s experiences

The researcher (NH) attended 16 antenatal clinics over a two month period, and approached all eligible women in attendance to discuss the study (n = 31). Twenty two women agreed to participate (71%), and 17 were interviewed (55%) (three women withdrew prior to interview and two failed to attend). The majority of women interviewed were in their 3^rd^ trimester (Table 1). Interviews were carried out at the participants homes, and using private rooms at Sure Start Children’s Centres and at the maternity unit. Most women were interviewed on their own; one woman had her partner present, one had her mother present, and two had their children present.

**Table 1:** Study 1 Participant Details.

Pseudonym^1^	Care Pathway (BMI, kg/m^2^)	Parity	Stage of Pregnancy at Interview
Kerry	30.0–34.9	1	3rd trimester
Anna	30.0–34.9	0	3rd trimester
Hayley	30.0–34.9	1	2nd trimester
Ella	35.0–39.9	0	3rd trimester
Adele	35.0–39.9	0	2nd trimester
Carol	35.0–39.9	1	3rd trimester
Alex	35.0–39.9	0	3rd trimester
Sophie	35.0–39.9	1	3rd trimester
Emily	>40	1	3rd trimester
Denise	>40	0	3rd trimester
Grace	>40	1	3rd trimester
Olivia	>40	1	3rd trimester
Rachel	>40	1	3rd trimester
Charlotte	>40	2	2nd trimester
Leah	>40	1	3rd trimester
Julie	>40	1	3rd trimester
Zoe	>40	4	3rd trimester

^1^ The participant pseudonyms are randomly selected names to maintain anonymity of the research participants.

Approximately half of the participants were eligible to attend the healthy lifestyle clinic (BMI>40kg/m^2^), and only one eligible participant was not attending. This proportion broadly represents overall engagement with the clinic (a local audit carried out at the time of the study showed 94% of eligible women attended the midwifery component of the clinic for the duration of their pregnancy, and 91% also had regular consultation with the dietitian). The final framework themes and sub-themes were:

1. Overall views of the pathways
  - Positive views and experiences
  - Negative views and experiences
2. Women’s knowledge and understanding of the pathways
  - The use of BMI in the pathways
  - Being on the pathways
3. Women’s views on the advice and support provided
  - Clinical management advice and support
  - Weight management advice and support
4. Women’s views of the patient information leaflet
  - Awareness of the leaflet
  - Leaflet content

#### 1.Overall views of the pathways

*1a*. *Positive views and experiences*. There was a consensus view that the pathways were beneficial. Women felt reassured that healthcare professionals were concerned about their health and their baby. Healthcare professionals’ approach was described as being friendly, supportive, and understanding. Women attending the healthy lifestyle clinic were positive about the additional support and time to discuss concerns.

*“It’s brilliant*, *there’s always somebody who’ll sort of go ‘well actually you can do this*, *or this is how to help this’*, *I think they’ve been brilliant*…*It was all handled very sensitively*, *it was all put across nicely and clearly*…*it’s very positive*, *it doesn’t sort of go ‘look fatty*, *you shouldn’t be doing that’*, *it’s just sort of* *help* *rather than*, *it’s friendly advice*, *it’s very positive”*

(Olivia, BMI>40kg/m^2^)

*1b*. *Negative views and experiences*: When women were not eligible for the healthy lifestyle clinic, the pathways were perceived to be a *“checklist”* which identified obesity-related risks but lacked weight-related support. These women wanted extra support to alleviate their concerns about risks. Some women received contradictory advice and messages about risk which was confusing and frustrating, and some had commenced the pathway in late stages of their pregnancy (rather than at booking).

*“I’m worried about* *this* [the pathway], *because they kept saying to me ‘your BMI is high’*…*(Anna’s Mother*: *I’ve been to every appointment with her they’re making a* *big issue* *with this BMI*, *but they’re* *not* *following it up with anything)*. *Yeah*, *they seem to be doing all the checks in here…*[maternity notes/pathway proforma] *they’re doing their job*, *but they’re not*, *coz they’re not giving me the things what I want*, *like the advice that I want*. *(Anna’s Mother*: *They’re making such an issue*, *every time we go it’s BMI*, *BMI*, *BMI*, *and then you’re constantly saying ‘well what do I do*…*to keep it safe*?*’)”*

(Anna, BMI 30–34.9kg/m^2^)

Women discussed negative associations with being classified as obese. Denise (BMI>40kg/m^2^) described a negative communication experience as being the *“only bad thing”* about the pathways: “*because she couldn’t get a good* [scan] *picture*, *I was just about to say ‘oh is it because I’m overweight*?*’*, *and the way she turned around and said ‘there’s obviously a lot of you to get through’*, *which I thought was the only bad thing*…*it was the way she said it*, *I mean she could have said like obviously ‘it’s harder’*, *but she just said ‘there’s a lot of you to get through’*”. The majority of women considered the negative associations of obesity to be a minor issue in comparison with the benefits of being on the pathway.

*“With anything in life*, *once you’re sort of in a category it’s almost well that becomes who you are…that would be the only negative that I would see*…*its human nature isn’t it*? *You tend to get stereotyped*, *and then once you’re given that stereotype it’s very difficult for people to think of you in any other way*. *You know so I think just from* *that* *side it’s not such a good thing*. *But I think it’s a very* *small* *thing”*

(Carol, BMI 35–39.9kg/m^2^).

There were also financial barriers to women attending multiple hospital appointments associated with the pathways (e.g. car parking costs), and time barriers when women worked or had children.

#### 2.Women’s knowledge and understanding of the pathways

*2a*. *The use of BMI in the pathways*: Most women remembered having their weight measured, BMI calculated, and understood that BMI classified their weight status. They viewed these procedures as *“routine”* and *“expected”*. However, women’s understanding of why their BMI was required was variable. Some felt they definitely had or had not received an explanation from their healthcare professional about the use of their BMI. Additionally, some women could not remember if they had received an explanation for a number of reasons, including the length of time since their first antenatal appointment, the amount of information they received, or when other priorities took precedence (e.g. illness) limiting their ability to retain all information provided.

“*Nothing much really* [was explained] *just that I need to check my weight because of my BMI*…*they need to just keep an eye on my BMI I think that was it really*, *all they said*…*I know I have to get weighed and stuff like that*, *that’s just routine*…*I don’t know* *why* *they needed to do it*, *that could be explained a bit better*”

(Charlotte, BMI>40kg/m^2^)

*2b*. *Being on the pathways*: Most women were aware of the pathway filed in their notes, and the few women who could not recall seeing the pathway had a BMI<40kg/m^2^. Women’s understanding of why they were on the pathways was variable, and those who did not understand wanted more explanation. Although some women felt they understood the need for the pathways, there was still some confusion and a lack of direct association between their weight and the additional intervention included in the pathways. When women felt they had a good explanation of the need for the pathways it increased their acceptance of the additional intervention.

*“It was put in* [the proforma put in the antenatal records], *and parts of it were filled in at the booking visit*, *it wasn’t really explained*…*it kind of confused me initially because obviously my BMI was 35 to 39 and I thought well what does that mean*? *That I’m high dependency when I give birth*? *Does that mean that if I put on loads of weight then I’m going to be further at risk*? *You know*, *I didn’t quite get it*…*If something’s being filled in and I don’t understand why then I kind of question why haven’t they told me*, *what are they hiding*?*”*

(Ella, BMI 35–39.9kg/m^2^)

*“I did think to begin with it was a little bit pointless*, *but as the midwife was going through what it was for with me I sort of understood a little bit that this was for our own good*…*because it just seemed like a lot of extra fuss for nothing*, *now I know it’s not for nothing*, *once I was informed of what it was for it was sort of like ‘oh okay then’*…*once somebody was telling me* *why* *it was happening”*

(Olivia, BMI>40kg/m^2^)

#### 3.Women’s views on the advice and support provided

*3a*. *Clinical management advice and support*: Overall, women were positive about the increased antenatal contact with healthcare professionals and found the extra monitoring and screening reassuring, particularly the additional scans.

*“I felt really reassured afterwards*, *I’d had a glucose tolerance test*…*and the result of that was all fine*, *so part of that was ‘Oh*!*’* [feeling good/relief] *I felt relieved everything’s alright there*…*she said that there shouldn’t be any major concerns because I didn’t have high blood pressure and I haven’t got diabetes”*

(Ella, BMI 35–39.9kg/m^2^)

However, some women described upsetting and frightening obesity-related risk communication which increased anxiety, particularly among nulliparous women in relation to the birth. Other women found the risk communication helpful even when they felt scared and actively sought information from their healthcare professionals. However, women often did not fully understand the explanation of risks from healthcare professionals and searched for additional information themselves. This was considered to be more frightening than receiving information from healthcare professionals. When women felt they had not received enough explanation they were internally amplifying the potential for risks and complications in the absence of this information.

*“It does frighten you coz I went home and sat and cried my eyes out when they’ve said to me ‘you’re gonna have a massive baby’*, *because I’ve panicked about how I’m gonna get her out”*

(Anna, BMI 30–34.9kg/m^2^)

*“Some more information about the birth and things like that*. *Not in general I mean if there’s anything specific to people that have been put on this pathway*, *because obviously they’ve said there* *could* *be complications*, *but no-one’s actually said what they might be*…*they could have said ‘there’s potential for this to happen*, *but this is what we’d do’ and maybe just a little bit of reassurance”*

(Adele, BMI 35–39.5kg/m^2^)

*3b*. *Weight management advice and support*: Women discussed weight management and lifestyle support more frequently than any other topic. Most women with a BMI<40kg/m^2^ felt their weight-related support was inadequate, and considered this an important omission from the pathways. Midwives provided general dietary information which women already knew, but they appreciated having their knowledge reinforced by a healthcare professional. However, physical activity was rarely discussed. Women felt that additional support would ease their worries about the impact of weight gain on them and their baby. Some women stated that they wanted a dietitian to provide personal and pregnancy-specific dietary advice rather than general information. In contrast, attendees of the healthy lifestyle clinic (BMI>40kg/m^2^) felt they received tailored dietary support which had influenced their weight management.

*“Obviously I’m on the BMI pathway but there hasn’t really been anything specifically* *for* *that if you know what I mean*…*I would have thought that because I’m on the pathway there would have been* *some* *kind of*…*appointment or a session or something*…*just a bit more information because it’s just kind of ‘get on with it’*, *but obviously you’re on this pathway anyway*, *it’s been identified*, *so I would have thought there’d have been something else*…*just maybe like ways of helping you keep your weight in control and that kind of thing*”

(Adele, BMI 35–39.9kg/m^2^)

Women expected, and wanted, to be weighed routinely during pregnancy. Those who were not eligible for the healthy lifestyle clinic were not routinely weighed following booking, and were surprised by the limited weight monitoring. Those attending the healthy lifestyle clinic were weighed regularly and used this monitoring to assess their progress. When women perceived that their weight gain was within a satisfactory range it had a positive impact on their motivation and self-esteem. Women regularly described positive weight gain reinforcement from healthcare professionals which also had a positive impact.

*“Yesterday it’s the first time I’ve been weighed since my booking appointment which I found a bit odd*…*I just assumed I don’t know obviously with the BMI thing being an issue I would have thought it was possibly something that they would have kept a check on*…*I suppose I would have* *wanted* *it in a way”*

(Alex, BMI 35–39.9kg/m^2^)

*“*[The dietitian was] *very pleased with my progress coz I’ve put on four or five pounds in the whole of my pregnancy*. *So that’s why I feel good about it as well*. *It’s proved that I* *can* *do it*, *not that I have to do it*, *I* *want* *to do it”*

(Rachel, BMI>40kg/m^2^)

Some women described their concerns about losing weight postnatally, were motivated to lose pregnancy weight gain, and wanted postnatal support. More focus was given to postnatal discussions among healthy lifestyle clinic attendees.

*“Just getting more information it’s made me* *think* *about losing weight* [postnatally]. *I mean I was gonna do it anyway before I fell pregnant*. *But now I’m determined because it’s not very nice being overweight and pregnant*…*I’m definitely doing it*!*”*

(Denise, BMI>40kg/m^2^)

#### 4. Women’s views of the patient information leaflet

*4a*. *Awareness of the leaflet*: There were equal proportions of women who had seen the leaflet, had not seen it, and those who were unsure. Some women questioned the usefulness of the leaflet due to the number leaflets they received during pregnancy. Additionally, the messages in the leaflet made behaviour change appear too simplistic and verbal interaction with healthcare professionals was preferred. However, others felt that leaflets were useful reference materials which helped with their behaviours, and those who had not previously seen the leaflet wanted to have received the information earlier in their pregnancy.

*“I’d like to go somewhere and* *do* *something yeah*, *because leaflets*, *you read them*, *you bin them*, *you don’t read them anymore*, *you don’t even remember what they said*, *whereas if you go and* *do* *it*, *it’s you know* [it’s more helpful]*”*

(Hayley, BMI 30–34.9kg/m^2^)

*4b*. *Leaflet content*: Women felt that the content on managing weight gain to reduce risks was new information. Most hadn’t realised the extent of the risks before reading the leaflet, and found this information helpful, motivating, and wanted further information about minimising risk. Women found the practical lifestyle tips relating to diet and activity to be helpful. However, the majority viewed the lifestyle information to be very basic, common sense information, a repetition of other information sources, and wanted more content on pregnancy-specific benefits. Women also wanted more information about how to access support services and further information.

*“The bit about exercise is quite good because I’ve read things that say obviously keep active and everything*, *but not specifically that says try and do 20 minutes a day or whatever…there’s a few bits in there that I can see just looking quickly that I might not have* *thought* *about*, *like taking a packed lunch if I was more organised*, *so there are some good tips…just like little hints and tips that make you think ‘oh actually that’s a good idea actually*, *I could do that’”*

(Adele, BMI 35–39.9kg/m^2^)

*“I was looking for like antenatal swimming and stuff like that*, *but there’s not a lot of it about or certainly I couldn’t find a lot you know…Maybe some information about where you can get that from*…*something a bit more concise that you could go to specifically to find out all the information around for antenatal stuff”*

(Alex, BMI 35–39.9kg/m^2^)

### Study 2 results: survey research exploring healthcare professionals’ experiences of delivering the clinical pathways

The questionnaire was mailed to all healthcare professionals responsible for delivering the pathways within the NHS Trust (n = 243; 86% midwives and 14% medical clinicians), and there was 68% response. The highest response was from community midwives (92%), and the lowest from hospital-based midwives (57%). The response rate from medical clinicians ranged from 83–87%. Therefore all disciplines apart from hospital-based midwives exceeded the target 70% response rate. The only significant difference in results between clinicians and midwives related to training needs (section 8). Therefore results for all healthcare professionals have been combined, except for section 8 where results are presented for midwives and clinicians separately.

The statistical analyses are presented in Table 2 which directly links the numerical codes for each statement with the questionnaire and the associated discussion of results (e.g. 4a in Table 2 relates to the statement labelled 4a in the questionnaire [see S1 Questionnaire], and to the label 4a cited in the discussion of results). In respondent provided 645 qualitative comments, with a similar proportion to the population sample (81% of comments were from midwives, 19% from clinicians). These comments were integrated with the quantitative data. Results originating from qualitative data are labelled QUAL in the discussion of results, and direct quotations from the questionnaires have been included.

**Table 2:** Component Study 2 Survey Results.

Questionnaire section numbers and statements	Mean (SD)	1 Strongly Agree (%)	2 Agree (%)	3 Neither Agree or Disagree (%)	4 Disagree (%)	5 Strongly Disagree (%)	Mean Result
4a: I know why the maternal obesity pathways have been implemented	1.6 (0.6)	41.4	56.6	1.4	0.7	0.0	Agree
4b: I don't know why there are different pathways for different obesity groups	3.8 (1.0)	3.6	12.2	7.2	56.1	20.9	Disagree
4c: I agree with the BMI cut offs used in the pathways	2.3 (0.7)	10.5	54.5	29.4	5.6	0.0	Agree
4d: Maternal obesity is an important clinical issue in pregnancy	1.4 (0.6)	58.6	40.0	0.7	0.0	0.7	Strongly Agree
4e: Maternal obesity is an important social issue in pregnancy	1.7 (0.7)	43.4	46.9	7.6	2.1	0.0	Agree
4f: Maternal obesity is a public health priority rather than an issue for maternity services	2.7 (1.1)	14.7	30.1	26.6	26.6	2.1	Neither Agree or Disagree
4g: I agree with the content of the pathways	2.1 (0.6)	13.4	63.4	21.8	1.4	0.0	Agree
4h: I would change some content of the pathways	3.3 (0.8)	2.5	14.2	41.7	39.2	2.5	Neither Agree or Disagree
5a: I find it difficult to discuss BMI with obese pregnant women	3.3 (1.0)	3.5	25.0	16.7	46.5	8.3	Neither Agree or Disagree
5b: I am more confident discussing the maternal obesity risk since the pathways were implemented	2.4 (0.7)	4.8	57.8	27.9	8.8	0.7	Agree
5c: I am less confident in discussing BMI status with patients since the implementation of the pathways	3.8 (0.7)	0.7	1.4	23.8	62.2	11.9	Disagree
5d: I am more confident in giving weight gain advice to obese women since the pathways implementation	2.6 (0.7)	4.9	38.2	45.8	11.1	0.0	Neither Agree or Disagree
5e: I am confused about the weight gain advice I should be giving to women on the obesity pathways	3.3 (0.9)	2.8	18.9	26.6	45.5	6.3	Neither Agree or Disagree
5f: I don't feel qualified to discuss obesity with pregnant women	3.6 (0.9)	1.4	14.8	14.1	59.9	9.9	Disagree
6a: There is an improvement in multi-disciplinary care since the pathways were implemented	2.6 (0.8)	4.3	50.0	34.8	8.0	2.9	Neither Agree or Disagree
6b: I see the benefits in having maternal obesity pathways	2.1 (0.6)	11.6	75.3	9.6	2.7	0.7	Agree
6c: There are more disadvantages to the pathways than benefits	3.8 (0.6)	0.0	1.5	29.2	60.6	8.8	Disagree
6d: The pathways are cost effective	2.9 (0.6)	1.4	20.3	70.3	8.0	0.0	Neither Agree or Disagree
6e: The facilities don't always allow compliance with the pathways	2.9 (0.9)	6.6	28.5	40.9	22.6	1.5	Neither Agree or Disagree
6f: I think the pathways could be better	3.2 (0.6)	1.6	8.5	62.8	26.4	0.8	Neither Agree or Disagree
7a: Discussing obesity upsets women	2.9 (0.9)	2.1	37.9	31.4	28.6	0.0	Neither Agree or Disagree
7b: I have experienced positive feedback from patients when I have discussed the obesity pathways	2.8 (0.8)	3.5	30.3	49.3	16.9	0.0	Neither Agree or Disagree
7c: Women are receptive to weight control advice in pregnancy	2.7 (0.8)	0.7	46.0	32.4	20.9	0.0	Neither Agree or Disagree
7d: Women don't understand why they are on the pathways	3.3 (0.9)	1.4	21.8	27.5	48.6	0.7	Neither Agree or Disagree
7e: Women don't accept they are obese	2.9 (0.9)	5.0	30.9	32.4	31.7	0.0	Neither Agree or Disagree
7f: Women are compliant with the pathways during pregnancy	3.0 (0.8)	0.7	25.5	45.4	27.7	0.7	Neither Agree or Disagree
8a: I don't feel that I need any training for this issue	3.6 (0.9)	0.7	16.5	17.3	53.2	12.2	Disagree
8b: I would benefit from some training around obesity in general	2.3 (0.8)	9.6	64.4	14.4	11.0	0.7	Agree
8c: I would benefit from some training about the risks of maternal obesity	2.5 (1.0)	7.9	58.6	11.4	20.7	1.4	Agree
8d: I would benefit from some training about weight gain advice for obese women in pregnancy	2.2 (0.8)	9.4	71.0	10.1	8.7	0.7	Agree
8e: I would benefit from some training about the safety of dieting in pregnancy	2.3 (0.8)	10.0	68.6	7.1	13.6	0.7	Agree
8f: I would benefit from some training about the safety of exercising in pregnancy	2.5 (0.9)	7.9	57.9	12.1	21.4	0.7	Agree
8g: I would benefit from training around sensitively discussing the issue of obesity with women	2.4 (1.0)	13.6	51.4	13.6	20.7	0.7	Agree
8h: I would benefit from some training but I'm not sure what in	3.4 (0.9)	1.6	15.1	38.9	35.7	8.7	Neither Agree or Disagree
9c: The information leaflet has not been useful to me^1^	3.4 (0.8)	0.0	13.2	36.8	47.4	2.6	Neither Agree or Disagree
9d: The leaflet has helped me raise the issue of maternal obesity with women^1^	2.5 (0.8)	5.3	50.0	31.6	13.2	0.0	Agree
9e. There is not enough information on the leaflet^1^	3.3 (0.7)	0.0	13.9	47.2	38.9	0.0	Neither Agree or Disagree
9f. The leaflet is easy to follow^1^	2.3 (0.6)	2.7	67.6	27.0	2.7	0.0	Agree
9g. The leaflet could be better^1^	3.1 (0.6)	0.0	11.4	68.6	20.0	0.0	Neither Agree or Disagree
9h. The information in the leaflet is appropriate^1^	2.4 (0.5)	2.9	58.8	38.2	0.0	0.0	Agree

^1^ Results only from healthcare professionals who had seen the leaflet (determined by question 9a).

#### Questionnaire Sections 1–4: Awareness, knowledge and understanding of the pathways

Overall awareness of the pathways was high among respondents (average 90%): 89–100% among obstetricians and midwives; 60–69% among anaesthetists and trainee obstetricians. The remaining results only include data from healthcare professionals who were aware of the pathways.

Most healthcare professionals knew that three pathways had been implemented (70%), although there was less knowledge of the correct BMI criteria used to differentiate the three (57%). Healthcare professionals knew why the pathways were in place and agreed with the current BMI criteria, although some considered that using BMI to *“categorise women”* was too rigid, and that individual assessments should be prioritised (4a-c, QUAL). There was also agreement with the content of the pathways, although some suggested developing pathways for women with *“very serious”* risks (e.g. BMI>50kg/m^2^) and reducing the *“lower risk”* content (e.g. screening and assessments) (4g, QUAL).

*“Otherwise healthy (borderline) women fear their pregnancy is at risk*. *Need to apply GUIDELINES appropriately on an individual base*.*”*

(Hospital-based midwife)

*“I find protocols inhibit good clinical care in a number of ways*. *A single BMI figure is generally helpful*, *but fat distribution and health are more important”*

(Consultant obstetrician)

Maternal obesity was considered to be an important socio-medical issue requiring integration of public health and maternity services (4d-f, QUAL). There were views that obesity should be addressed in pregnancy due to risks and complications, that pregnancy was a good time to tackle public health issues, and the need for more public health messages on the impact of obesity on pregnancy (QUAL).

*“I think the general public should have a greater understanding of risks of high BMI in pregnancy*, *very little knowledge available”*

(Community midwife)

*“They all know they are overweight and that that risks their health*. *They are less aware of risks for their babies”*

(Consultant obstetrician)

#### Questionnaire Section 5: Confidence

Although healthcare professionals neither agreed nor disagreed that it was difficult to discuss obesity (5a), extensive qualitative data were provided on communication barriers. These included obesity-associated stigma, sensitivity, making women feel uncomfortable, feeling judgemental, feeling overly negative, limiting women’s choices, and healthcare professionals having their own weight issues (QUAL).

*“I feel uncomfortable discussing these issues when obese women are already VERY self-conscious and upset by their weight also when we are trying to build a non-judgement relationship with them”*

(Community midwife)

*“I find obesity difficult to discuss as I feel it is sometimes a sensitive issue*.*”*

(Hospital-based midwife)

Some healthcare professionals felt that discussing lifestyle was part of routine care for all women, whereas others perceived general advice to be insufficient due to the complexity of obesity (QUAL). There was also a consistent view that obesity discussions were easier with some women than others, and descriptions of strategies employed to facilitate discussions such as using terminology deemed to be least offensive and promoting benefits to the baby (QUAL). The perceived variability in ease of obesity discussions could potentially explain the central tendency to question 5a rather than a lack of strong views in either direction. Healthcare professionals felt that having the pathways in place provided defined categories which supported discussions, and the focus on clinical requirements increased their confidence to discuss risks (5b, QUAL).

*“I feel they have been a good idea and made it easier for us to raise the issue with our clients—giving them facts without appearing judgemental and offering praise and support”*

(Community midwife)

There was a central tendency to neither agree nor disagree with questions on weight gain confidence and confusion (5d-e). Some healthcare professionals felt they had always been confident discussing weight gain with pregnant women, whereas for others this was the most confusing aspect of the pathways with which they had least confidence (QUAL). These healthcare professionals questioned what advice they should give, what to do if weight gain was excessive, and felt there was a lack of guidance on these issues.

*“Weight gain advice has been difficult to get a straight answer*!*”*

(Community midwife)

*“I would like more information re weight gain that is acceptable”*

(Community midwife)

#### Questionnaire Section 6: Worthwhile

Overall, the pathways were viewed as being beneficial, provided structure, and filing proformas in women’s antenatal records facilitated implementation and discussion (6b-c, QUAL). Implementation was also considered to be dependent on women’s motivation, knowledge, and socio-economic status (QUAL). Barriers to implementation were deemed to be inconsistency between individual healthcare professionals compliance, missing or incomplete pathways in antenatal records, when advice from healthcare professionals contradicted the pathways, and limited time in routine contacts for assessments and screening (QUAL). Community midwives particularly described time limitations to discuss lifestyle in a sensitive and supportive way which addressed complex needs (QUAL).

*“I think the pathways are very good and go a long way to encouraging the women to take caution of their weight problem*. *The fact that they are in the notes is a positive*, *and women can refer to them and know they are receiving appropriate care”*

(Hospital-based midwife)

*“*[Discussing healthy lifestyles] *we all do this for all women but obese women obviously need more time devoted to this as they obviously have an issue and need to be educated more*. *Time limitations make it difficult”*

(Community midwife)

*“There still seems to be confusion amongst work colleagues which pathway these women should be on”*

(Community midwife)

Healthcare professionals neither agreed nor disagreed with statements relating to cost-effectiveness, supporting facilities, and whether the pathways could be improved (6d-f). However, they described difficulties accessing services (e.g. dietetics), vitamin D supplements, and equipment (e.g. appropriate scales, blood pressure cuffs and TED stockings) (QUAL). Recommendations for improvements also had a strong focus on supporting women with the provision of more information, weight management, and lifestyle support. There were suggestions for the pathways to address socio-economic issues (e.g. free or subsidised services), to involve women in future pathway development, and for collaborative work with primary care and community services to facilitate pre-conception, pregnancy, and postnatal support (QUAL).

*“Perhaps women who are on these pathways should be asked their opinions as to how to effect and manage their particular situation”*

(Hospital-based midwife)

*“Continuity of care with community dietetic support*, *exercise programme*, *postnatally”*

(Hospital-based midwife)

#### Questionnaire Section 7: Response from women

There was a central tendency to neither agree nor disagree with all of the statements relating to responses from women (7a-f). However, this topic was the most frequently discussed by healthcare professionals, repeatedly throughout different questionnaire sections (QUAL). Healthcare professionals felt that the variability in women’s response made it difficult to dichotomise into agreement or disagreement with the statements.

*“All of this* [response from women] *entirely depends on character*, *circumstances and reasons for obesity*. *Cannot totally be answered by a tick”*

(Community midwife)

*“Different women react differently depending on their own body image”*

(Community midwife)

The difference in women’s response was felt to be dependent on two primary factors: the individual woman; and the healthcare professionals’ approach (QUAL). Weight-related terminology was considered to influence women’s responses, with the terms *“obese”* and *“fat”* evoking negative reactions. Healthcare professionals predominantly avoided these terms, although sometimes used obese when discussing risks. Women’s negative responses to discussion were described as “*upset”*, *“embarrassed”*, *“low self-esteem”*, *“guilt”*, *“defeatist” and “defensive”*. However, women’s positive and proactive responses were described proportionately to negative, and some women were perceived to be relieved to discuss their weight. The terms *“overweight”* and *“raised BMI”* were felt to be more positive and most frequently used by healthcare professionals.

*“Obesity is usually used in prejudiced sense”*

(Community midwife)

*“Labelling them "obese" in notes can upset patients and make them lose temper sometimes”*

(Trainee obstetrician)

*“I use BMI—I do not use the term obesity as some women find this offensive”*

(Consultant obstetrician)

Positive responses were most frequent when women were already aware of their weight status, they had a good rapport with healthcare professionals, once they understood the need for the pathways, and when the potential benefits for the baby were emphasised. These negative and positive reactions were also considered to influence how receptive women were to the advice provided (QUAL).

*“If I already know the woman they respond well to advice re weight gain and being weighed”*

(Community midwife)

*“Most women know if they are obese*, *and if discussed sensitively and appropriately do try to eat healthily in pregnancy”*

(Community midwife)

*“Generally very well* [response from women]. *When we discuss the risks involved*, *they understand why we are discussing this”*

(Consultant obstetrician)

*“Most women are aware they are overweight if they are*! *Some are embarrassed*, *most seem willing to listen to advice and the baby can be an incentive for healthy changes*.*”*

(Community midwife)

Despite healthcare professionals neither agreeing nor disagreeing with the statement about women’s understanding of being on the pathway (7d), some felt that women did not perceive their weight as a risk factor, recognise birth choice limitations, or were in denial of their obesity (QUAL). Others felt that women were aware of their weight status, and acceptance of this influenced women’s compliance with the pathways.

*“Some women are happy to discuss obesity and receptive to weight control advice*. *These women appear to accept that they are obese*. *Others deny obesity and can become upset*.*”*

(Community midwife)

*“Some accept they are large ladies*, *others have got upset*, *denying they are big and saying it has spoilt their pregnancy experience*.*”*

(Community midwife)

*“Obesity is a major issue not just in pregnancy and many patients either do not see it as a problem or prefer to ignore the consequences”*

(Hospital-based midwife)

Healthcare professionals frequently stated that women were compliant with the clinical aspects of the pathways, but were reluctant to engage with weight management (QUAL).

*“Very few women in my experience wish to be referred to a dietitian and find it very difficult to discuss weight issues*. *The higher the BMI the more women don’t wish to discuss weight”*

(Community midwife)

*“They don't like being re-weighed and avoid discussing weight gain*!*”*

(Consultant obstetrician)

*“Women readily accept that a medical condition such as hypothyroid causes their obesity—so thyroid tests are easy*… *Diet is the most difficult aspect*. *They will readily accept extra scans”*

(Community midwife)

#### Questionnaire Section 8: Training

The majority of healthcare professionals felt that they did require training, and agreed with all of the statements relating to topic specific training (8a-g). There were no significant differences between medical clinicians and midwives in their agreement about requiring training (8a p = 0.12), or relating to training on sensitive discussions (8g p = 0.08). However, midwives were more likely than clinicians to agree they required training on obesity in general and maternal obesity risks (8b-c: p<0.001), weight gain (8d: p = 0.005), and the safety of dieting and exercise in pregnancy (8e-f: p = 0.003).

Despite the overall agreement for requiring training, some healthcare professionals described their existing knowledge or training on specific topics as justification for not requiring additional training (8a, QUAL). However, the majority of discussion emphasised the benefits of having up-to-date evidence-based training in order to: improve knowledge and patient care; have an impact on women’s behaviours; and promote consistent practice. An additional training topic on accessing support services was added to the pre-defined list by some respondents (QUAL).

*“Community midwives are involved with women from the very start of their pregnancy*. *If better trained they could have more impact”*

(Community midwife)

*“Always good to have up to date info and be aware of acceptable weight gains in all BMI pathways”*

(Community midwife)

*“*[Don’t require additional training] *Have already arranged to attend an Association of Anaesthetists study day on obesity related anaesthesia issues”*

(Consultant anaesthetist)

#### Questionnaire Section 9: Leaflets

There was limited awareness of the healthcare professional leaflet (clinicians 32%, midwives 30%). Among those who were aware, midwives were more likely than clinicians to use it in practice (78% versus 57%). The majority found it easy to follow, felt that the information was appropriate and helped them raise the issue with women (9dfh). Healthcare professionals’ views on appropriate leaflet content for women primarily related to lifestyle and weight-related advice, and signposting to available support (QUAL). Risks and implications for labour choice were also considered appropriate.

*“A positive approach with ideas on how to change their eating habits or ways they prepare food to make them more healthy”*

(Community midwife)

There were similarities in healthcare professionals’ perspectives on appropriate leaflet content to support their practice. However, they felt some additional information was required on approaching discussions and weight gain (QUAL).

*“How to approach discussing obesity with women*. *What amount* [of weight] *is acceptable to gain and what to do if they gain more”*

(Community midwife)

### Study 3 results: clinical audit of compliance with care pathway 3 (BMI≥40kg/m^2^)

The antenatal records for 60 randomly selected women with a booking BMI>40kg/m^2^ were retrieved. One record was excluded due to a first trimester miscarriage. The results compare antenatal, intrapartum, and postnatal standards of care within the pathways (Table 3). Overall, 31 (67%) standards of care were compliant (Table 3). There was full compliance for seven standards, and a further eight standards with over 90% compliance. However, compliance was less than 50% for seven standards of care, the lowest being the provision of postnatal dietetic support (17%) and healthy lifestyle advice (29%).

**Table 3:** Component Study 3 Audit Results.

Contact	Audit Standard of Care on Pathway	Compliance (%)	NHSLA Criteria: Pass (≥75%); Fail (<75%)
**Antenatal mean compliance 75%**			
**Booking**	Calculate BMI	100	Pass
	Referral: high dependency ANC	100	Pass
	Referred to obstetric medical clinic^1^	100	Pass
	Offer thyroid function	90	Pass
	Explain obesity implications	85	Pass
	Offer Folic acid	85	Pass
	Discuss weight management	83	Pass
	Offer GDM screening	81	Pass
	Provide leaflet	78	Pass
	Give exercise advice	78	Pass
	Offer Vitamin D	71	Fail
**High Dependency Antenatal Clinic**	Plan on-going AN care	92	Pass
	Complete alert card	90	Pass
	Book anaesthetic review	88	Pass
	Offer community dietetics referral^2^	83	Pass
	Discuss risks	81	Pass
	Discuss weight management/exercise	76	Pass
**On-going AN Care**	USS at 32wks	86	Pass
	USS at 36wks	83	Pass
	Appropriate BP cuff	54	Fail
	Weight at 32wks	51	Fail
	Anaesthetic review^3^	50	Fail
	Weight at 28wks	48	Fail
	Continue encouragement re: diet/activity	37	Fail
	Manual handling risk assessment	34	Fail
**Antenatal Admission >24hrs** ^**4**^	Liaise with midwife/clinician for induction of labour	100	Pass
	Thromboembolism risk assessment	33	Fail
**Intrapartum mean compliance 82%**			
**Intrapartum**	Did not labour on low dependency labour ward	100	Pass
	CS decision at consultant level—documented^5^	100	Pass
	Consultant anaesthetist informed (if surgery anticipated) ^5^	100	Pass
	FBC, group and save^5^	98	Pass
	Continuous CTG/FSE monitoring^5^	90	Pass
	Ranitidine as per protocol^5^	90	Pass
	Pressure care guideline^5^	90	Pass
	Consultant present for LSCS ^5^	65	Fail
	Consultant obstetrician involved^5^	62	Fail
	Inform duty anaesthetist^5^	58	Fail
	TED stocking in labour^5^	45	Fail
**Postnatal mean compliance 67%**			
**Postnatal**	Postpartum thromboprophylaxis given	83	Pass
	Right dosage of thromboprophylaxis^6^	98	Pass
	Early ambulation encouraged	83	Pass
	Strict attention to wound and perineal care	83	Pass
	Contraception advice	75	Pass
	Breast feeding support	56	Fail
	Healthy life style advice	29	Fail
	On-going support from community dietetics services^7^	17	Fail

^1^Percent of applicable women for referral: with diagnosed diabetes mellitus or abnormal blood glucose.

^2^The pathways included a referral to community dietetics at the time of audit as the antenatal healthy lifestyle clinic was not in place.

^3^Percent of applicable women for review: with BMI>40kg/m^2^ and 1 diagnosed co-morbidity.

^4^Percent of women with antenatal admission >24hrs.

^5^Percent where applicable based on mode of delivery.

^6^Percent with the right dosage among women prescribed thromboprophylaxis.

^7^The pathways included a referral to community dietetics at the time of audit as the healthy lifestyle clinic was not in place.

Stratified by the stage of pregnancy, there was acceptable compliance for 19 out of 27 antenatal standards of care (70%), seven out of 11 intrapartum (64%), and five out of eight postnatal (63%). However, the mean percentage compliance for each stage of pregnancy showed an overall acceptable compliance for antenatal (75%) and intrapartum stages (82%), but not for postnatal (67%) (Table 3).

Within the antenatal standards of care, compliance was greatest at booking and in the high dependency antenatal clinic. Compliance was poor with offering vitamin D at booking, with all on-going antenatal care standards except ultrasound scans, and with thromboembolism risk assessment when women were admitted for more than 24 hours. Compliance with the majority of intrapartum standards of care was very good (>90%), with the exception of aspects of the pathways relating to the involvement of consultant obstetricians and anaesthetists, and the use of TED stockings in labour. Most of the postnatal standards of care with acceptable compliance related to clinical management, and poor compliance related to the provision of advice and support. None of the postnatal standards reached 100% compliance.

### Integration of results: studies 1, 2, and 3

The integration of research studies identified three meta-themes: overall views of the pathways, communication of the pathways, and content of the pathways. The integration focuses on the level of convergence, complementarity, dissonance and silence between studies within the meta-themes.

#### Meta-theme 1: Overall views of the pathways

There was agreement (convergence) between healthcare professionals and women that the pathways were beneficial (Table 4). However, there were different perspectives between populations relating to the nature of the benefits, and to the overall focus given to specific topics (dissonance). Women primarily viewed the benefits of the pathways to be the provision of additional support, and most frequently discussed weight management issues. However, healthcare professionals primarily viewed the benefits to be the provision of structure and focus to their practice, and most frequently described the responses from pregnant women on the pathways. Therefore there was dissonance between healthcare professionals’ and women’s views on the pathway’s key issues.

**Table 4:** Integration of Results: Convergence Coding Matrix for Meta-theme 1. Overall views of the pathways.

Meta-subtheme	1. Interviews with women (QUAL)	2. Healthcare professional questionnaire (QUANT + QUAL)	Convergence assessment
Overall view	(Theme 1) Women’s majority view was that the pathways were positive and beneficial.	(Section 6) The majority of healthcare professionals agreed that the pathway had benefits (Q6b) and disagreed that the pathways had more disadvantages than benefits (Q6c).	Convergence
Reasons for viewpoint	(Theme 1) Consistent view among women related to SUPPORT: the primary positive aspects were reassurance, and feeling that healthcare professionals were concerned about them and their babies.	(Section 6) Consistent view among healthcare professionals related to PRACTICE: the primary benefits were providing a structured approach, facilitating discussion, and supporting referrals for clinical management.	Dissonance
Most frequent reference to…	(Theme 3) Weight management/lifestyle advice	(Section 6) Responses from women	Dissonance

#### Meta-theme 2: Communication of the pathways

The results of the integration of data relating to the communication of the pathways (meta-theme 2 and all subthemes within) are presented in Table 5.

**Table 5:** Integration of Results: Convergence Coding Matrix for Meta-theme 2. Communication of the pathways.

Meta-subtheme	1.Interviews with women (QUAL)	2.Healthcare professional questionnaire (QUANT + QUAL)	3.Clinical audit: Pass (≥75%) Fail (<75%) (QUANT)	Convergence assessment
1: Healthcare professionals communication in practice	(Theme 1) Confusion and frustration when they received contradictory advice/messages.	(Section 6) Variation in healthcare professionals’ compliance, sometimes advice contradicted the pathways. (Section 8) Training would promote consistent practice.	Not applicable	Convergence
	(Theme 1) Pathways commenced at late stages in some women’s pregnancies.	(Sections 1–3) Variable awareness of the pathways between specialities (Q1), limited awareness of the BMI criteria (57%).	Not applicable	Complementarity
2: Women’s awareness and understanding of the pathways	(Theme 2) Variable recognition of the pathways being in their notes [BMI>40kg/m^2^ more aware]	(Section 6) Having pathways in women’s notes facilitated implementation, but pathways often missing or incomplete.	Not applicable	Complementarity
	(Theme 2) Variable understanding about the relationship between BMI and pregnancy, and recollection of explanations from healthcare professionals.	Not described	Pass: explaining obesity implications at booking	Dissonance
	(Theme 2) Variable understanding of the link between the pathway content and weight status. Understanding based on explanations received or women’s interpretation in the absence of explanations.	(Section 4) Healthcare professionals agreed that they knew why the pathways had been implemented (Q4a), and disagreed that they didn’t know why there were different pathways for different obesity groups (Q4b).	Not applicable	Dissonance
3: HCPs approach to communication	(Theme 1) Majority response positive to healthcare professionals approach: approach was friendly, supportive, understanding and approachable.	(Section 5) Barriers most frequently described: healthcare professionals agreed that they would benefit from training on sensitive discussion (Q8g). No difference between midwives and medics (p = 0.08).	Not applicable	Dissonance
	(Theme 1) Majority response positive to healthcare professionals approach: approach was friendly, supportive, understanding and approachable.	(Section 5) Strategies to facilitate positive discussions were described.	Not applicable	Complementarity
	(Theme 1) Minority response negative communication: negativity of being categorised as obese.	(Section 5) Barriers to discussing obesity were largely influenced by women’s responses. (Section 7) Women’s responses were proportionately positive and negative, and accepting or in denial.	Not applicable	Dissonance
	(Theme 1) One negative experience relating to obesity communication *“a lot of you to get through”*.	(Section 7) Terminology can influence women’s response: ‘obese’ and ‘fat’ (negative responses, predominantly avoided); ‘overweight’ and ‘raised BMI’ (positive responses, most frequently used).	Not applicable	Convergence
4: Risk communication	(Theme 3) Some women didn’t understand explanations of risks.	(Section 7) Women have limited recognition of pregnancy risk factors/birth choice limitations.	Not applicable	Convergence
	(Theme 2 and 3) Women want more detailed explanation/understanding of risks; how risks are managed; and explanations by healthcare professionals. The absence of explanation increased anxiety and searching for information was frightening.	(Section 5) Agreed they were more confident in risk communication (Q5b), disagreed they were less confident (Q5c), described how defined categories made discussions easier, more supportive, and non-judgemental. Barriers to risk communication are feeling overly negative or limit choices for women (Section 5). Midwives want training on risks (Q8c).	Pass: discussing risks in the high dependency antenatal clinic	Dissonance (studies 1 and 2/3); Complementarity (studies 2 and 3)
	(Theme 2) Increased understanding impacted on the acceptance of additional intervention.	(Section 7) When women understand benefits to them/their babies they are more receptive to advice.	Not applicable	Convergence
5: Emotional responses to communication	(Theme 3) Risk communication was the only consistent emotional response to communication for women, anxiety levels increased when risks weren’t adequately explained, some had been frightened or upset by risk communication.	(Section 6) Obesity-associated sensitivity and stigma makes discussions difficult.	Not applicable	Dissonance
	(Theme 3) Risk communication was the only consistent emotional response to communication for women, anxiety levels increased when risks weren’t adequately explained, some had been frightened or upset by risk communication.	(Section 7) Negative responses to obesity discussion described (e.g. upset, guilt, low self-esteem).	Not applicable	Complementarity
	(Theme 3) Active searching for risk information from healthcare professionals. Positive response when explanations were adequate, even when women found risks scary.	(Section 7) Positive responses to obesity discussion described (e.g. positive with more understanding and emphasis on benefits to their baby).	Not applicable	Complementarity

*2a*. *Healthcare professional communication in practice*: Healthcare professionals and women agreed that there was variation in healthcare professional compliance with the pathways, which had an impact on communication (Table 5). Both populations felt that this led to contradictory messages, and impacted on the awareness of pathways among women and healthcare professionals. There was also agreement that addressing poor communication between individual healthcare professionals could facilitate more consistent practice and messages for women.

*2b*. *Understanding the pathways*: There was complementarity between women and healthcare professionals relating to problems with pathways being filed in women’s antenatal records, and dissonance was present between the two populations in relation to understanding the pathways (Table 5). Healthcare professionals felt that they understood the pathways and the audit showed good compliance with explaining obesity implications at booking. However, there was variability in women’s understanding and recollection of explanations. This could indicate a difference between healthcare professionals perception of communicating explanations to women, and women’s ability to process the information received or their satisfaction with the explanations provided. Alternatively the issue may be related to the timing of communication. The booking appointment is the contact where the pathways commence, and therefore an important time to explain the need for the pathways. However, due to the amount of information women received at this contact it may be necessary to confirm that women adequately understand the pathways at subsequent contacts.

*2c*. *Healthcare professionals approach to communication*: There was convergence, complementarity, and dissonance relating to healthcare professionals approach to communication (Table 5). Both populations agreed that there could be positive responses to obesity discussion, and acknowledged the influence of terminology on women’s response. However, there was dissonance between population perspectives of communication approaches. Healthcare professionals focussed on the barriers to communication due to negative responses from women. They also felt that positive and negative responses from women were proportionate, as was women’s acceptance or denial of their obesity. This perspective was not reflected by women, where the majority described healthcare professionals’ approach as being positive and supportive, and only a minority identified potential negativity. In this sample of women there was no apparent denial of obesity status.

*2d*. *Risk communication*: There was convergence and dissonance relating to risk communication (Table 5). Both populations agreed that some women had limited understanding of maternal obesity risks, and that acceptance of intervention was higher when risks are understood. However, there was dissonance between all three studies and risk communication. Overall healthcare professionals felt the pathways had increased their confidence with risk communication, and the audit identified good compliance with discussing risks in the high dependency antenatal clinic. Whereas women felt that they did not always understand explanations, and wanted more risk information from healthcare professionals. Healthcare professionals perceived there to be barriers to risk communication due to the potential negative impact on women, whereas it was the absence of satisfactory risk communication which increased women’s anxiety. Some of the dissonance may be explained as not all obese women meet the criteria to attend the high dependency antenatal clinic (where compliance with risk communication is acceptable), and compliance outside of this environment may not be as high. In addition there may be a difference between women’s and healthcare professionals’ perception of adequate risk communication.

*2e*. *Emotional responses to communication*: There was complementarity between populations about the potential for obesity discussions to be upsetting as well as positive (Table 5). However, there was dissonance between women’s and healthcare professionals’ perspective on the causal nature of the upset. Women consistently related the lack of explanation and understanding of potential risks to cause their upset; whereas healthcare professionals predominantly attributed upset to psychological factors associated with obesity, such as sensitivity and stigma.

#### Meta-theme 3: Content of the pathways

The results of the integration of data relating to the content of the pathways (meta-theme 3 and all subthemes and subgroups within) are presented in Table 6.

**Table 6:** Integration of Results: Convergence Coding Matrix for Meta-theme 3. Content of the Pathways.

Meta-subtheme	Subgroup	1.Interviews with women (QUAL)	2.Healthcare professional questionnaire (QUANT + QUAL)	3.Clinical audit: Pass (≥75%) Fail (<75%) (QUANT)	Convergence assessment
a: Clinical advice and support	Not applicable	(Theme 3) Increased antenatal contact with healthcare professionals was positive, and increased monitoring and screening reassuring.	(Section 4 and 6) Reduce the need for screening and assessments on lower BMI pathways due to clinic time barriers	Fail: Thromboembolism, anaesthetic, and manual handling assessments	Dissonance (studies 1 and 2); Complementarity (studies 2 and 3)
	Not applicable	(Theme 3) Increased antenatal contact with healthcare professionals was positive, and increased monitoring and screening reassuring.	(Section 7) Women were compliant with the clinical aspects of the pathways. (Section 6) Implementation of the clinical aspects of the pathways was easy/positive.	Pass: Most clinical components at booking; high dependency clinic; on-going antenatal care, antenatal admission, and labour	Complementarity
	Not applicable	Not applicable	(Section 6) Difficulties in complying with some aspects of the pathways, due to limited access to equipment and resources (especially BP cuffs, TED stockings, and vitamin D).	Fail: Appropriate sized BP cuff, vitamin D at booking, TED stockings in labour	Convergence
b: Weight management and lifestyle advice and support	i. Lifestyle advice and support	(Theme 3) Adequate lifestyle advice/support among women attending the healthy lifestyle clinic, BMI>40kg/m^2^, but not among women with a BMI<40kg/m^2^. Midwives provide general advice, and the dietitian more personalised advice. Women want personalised pregnancy-specific support, and access to support services.	(Section 6) General lifestyle advice is routinely provided, but obesity is more complex. There is limited access to dietetic support. (Section 8) Midwives want training on dieting and exercise in pregnancy (Q8e&f), which would support women’s behaviours and access to support services.	Pass: discussing weight management/ lifestyle at booking and in the high dependency ANC.	Complementarity
	i. Lifestyle advice and support	(Theme 1) Additional time with healthcare professionals in the healthy lifestyle clinic in comparison with routine appointments was positive.	(Section 6) Limited time in routine appointments to discuss lifestyle and complex needs.	Fail: continued encouragement with diet and activity	Complementarity
	ii. Weight gain	(Theme 1) The pathways are a checklist process which emphasise risks of obesity, and lack support in addressing weight issues. (Theme 3) Weight gain advice is provided in the healthy lifestyle clinic, but not to women with a BMI<40kg/m^2^.	(Section 6) Limited access to weight management support (e.g. dietetics) needs to improve. (Section 5) Healthcare professionals neither agreed/disagreed that they were more confident/confused about weight gain advice (Q5d&e), but some described confusion/low confidence with this aspect, and the least guidance. (Section 8) Midwives want training on weight gain (Q8d), which would help them support obese women.	Not applicable	Complementarity
	ii. Weight gain	(Theme 3) Weight gain support was important due to concerns about their health risks, risks to the baby, and postnatal weight retention. Receiving weight gain support was positive (BMI>40kg/m^2^, healthy lifestyle clinic); not receiving it was negative (BMI<40kg/m^2^).	(Section 7) Perceived reluctance among women to engage with dietetic referrals and behaviour change advice for weight management.	Not applicable	Dissonance
c. Weight measurement and feedback	Not applicable	(Theme 2) Booking BMI acknowledged, expected, and viewed as being routine.	Not applicable	Pass: calculating BMI at booking	Convergence
	Not applicable	(Theme 3) Weight monitoring expected and wanted. Weight monitoring more likely at the healthy lifestyle clinic [BMI>40kg/m^2^].	(Section 6) Difficulty accessing appropriate weighing scales was a barrier to implementing the pathways.	Fail: weight monitoring at 28 and 32 weeks.	Complementarity
	Not applicable	(Theme 3) Variable level of weight gain feedback from healthcare professionals. Feedback was more likely among women attending the healthy lifestyle clinic (BMI>40kg/m^2^).	(Section 5) Lack of guidance on weight gain for obese women, unsure of appropriate advice and management of excessive weight gain.	Not applicable	Complementarity
	Not applicable	(Theme 3) Feedback was used to self-assess progress. Self-perceived satisfactory weight gain, and positive reinforcement from healthcare professionals positively impacted on motivation/self-esteem.	Not described	Not applicable	Silence
d. Postnatal support	Not applicable	(Theme 3) Postnatal weight management support was desired. Motivation for postnatal weight management was highest among those who had received dietetic support during pregnancy.	(Section 4) Healthcare professionals neither agreed/disagreed that maternal obesity was a public health priority rather than maternity (Q4f), and described it as an important socio-medical issue which required integration of public health and maternity services. (Section 6) More collaboration is needed between maternity services, community services, and primary care to support women preconception, in pregnancy and postnatally.	Pass: all postnatal clinical aspects of care and contraception advice; Fail: postnatal advice and support for breastfeeding, lifestyle, and dietetics services	Complementarity (studies 1 and 2); Dissonance (studies 2 and 3)
e. Time and monetary costs for women	Not applicable	(Theme 1) The main negative aspect of pathways was the time commitment required to attend multiple appointments. Some found this to be a significant issue (e.g. taking time off work, or organising childcare), others felt it was minor compared with the benefits.	Not described	Not applicable	Silence
	Not applicable	(Theme 1) There are cost implications for women to comply with the pathways due to multiple and lengthy hospital appointments.	(Section 6) The pathways should be improved to address SES issues (e.g. free/subsidised services).	Not applicable	Complementarity
f. Information Leaflets	i. Leaflet awareness	[Women’s leaflet] (Theme 4) Awareness was determined by BMI (BMI >40kg/m^2^ more aware)	[HCPs leaflet] (Section 9) Limited awareness (Q9a ~30% awareness; Q9b ~20% use in practice).	[Women’s leaflet] Pass: provide leaflet at booking	Complementarity (studies 1 and 3)
	ii. Usefulness of leaflets	(Theme 4) Divided opinion on the usefulness of leaflets: Positive views (useful reference material, helps change behaviours)	(Section 9) Healthcare professionals agreed that their leaflet helped raise the issue (Q9d), was easy to follow (Q9f), had appropriate information (Q9h)	Not applicable	Complementarity
	ii. Usefulness of leaflets	(Theme 4) Negative views (too many leaflets, simplistic view of behaviour, prefer interaction with healthcare professionals)	Not described	Not applicable	Silence
	iii. Leaflet content for women	(Theme 4) Reducing risks was new information, helpful, motivating. Some wanted more detailed risk explanation.	(Section 9) Leaflets for women’s use should include risks to mum and baby, and implications for labour choice.	Not applicable	Convergence
	iii. Leaflet content for women	(Theme 4) Lifestyle advice was general, they already knew it. Some practical tips were useful, wanted more information on pregnancy-specific benefits of nutrition and physical activity.	(Section 9) Leaflets for women’s use should include healthy lifestyle information which promotes benefits to women and families, practical advice, and motivators to change.	Not applicable	Convergence
	iii. Leaflet content for women	(Theme 4) Information on further information sources/support services wanted.	(Section 9) Leaflets for women’s use should signpost to available support.	Not applicable	Convergence
	iv. Priority of content	(Theme 4) Reducing risk: women’s view of the most useful content included in the leaflet	(Section 9) Lifestyle: healthcare professionals view of the most important content to include in leaflets for women	Not applicable	Dissonance
	v. Leaflet content for HCPs	Not applicable	(Section 9) Leaflets for healthcare professionals’ use should include similar information to leaflets for women, but more in depth information on risks, lifestyle support for women, and appropriate weight gain; and information appropriate to their practice (approaching discussion, appropriate weight gain advice, management of excessive weight gain)	Not applicable	Silence

*3a*. *Clinical advice and support*: There was convergence, complementarity, and dissonance between the three studies in relation to the provision of clinical advice and support (Table 6). There was agreement that women were positive about the clinical aspects of the pathways, and relating to the ease of implementation and compliance with most of these. However, healthcare professionals also identified barriers to implementing some clinical aspects due to restricted resources, which is convergent with the low compliance (e.g. appropriate sized TED stockings during labour). The dissonance within the context of this meta-theme is between healthcare professionals’ views that some screening and assessments should be reduced due to capacity, whereas women found the additional clinical intervention to be particularly positive, beneficial and reassuring.

*3b*. *Weight management advice and support*: There was complementarity and dissonance between the studies relating to the provision of weight management and lifestyle advice and support (Table 6). Healthcare professionals felt they provided general lifestyle advice to all women, they were compliant with this at booking, and women agreed that they received general advice. Both populations also felt that more tailored support was required from dietitians, which is supported by the difference in the level of support women felt they received based on their BMI and eligibility to attend the healthy lifestyle clinic. However, the majority of data related to barriers to providing weight gain and lifestyle support. Healthcare professionals’ description of multiple barriers is supported by low compliance with providing continued antenatal lifestyle encouragement. However, dissonance was present as healthcare professionals perceived that women were reluctant to engage with weight management advice and referrals, whereas women felt that this was an important aspect of their care which was missing from the pathways.

*3c*. *Weight measurement and feedback*: There was convergence, complementarity and silence between studies relating to weight measurement and feedback (Table 6). There was agreement between women acknowledging their BMI calculation at booking, and acceptable compliance with this. However, subsequent weight monitoring compliance was low, healthcare professionals described difficulties in accessing appropriate equipment, and women’s weight was only routinely monitored when they were eligible for the healthy lifestyle clinic. Similarly healthcare professionals described barriers to providing weight gain feedback, and only the women who were attending the healthy lifestyle clinic reported receiving weight gain feedback. Silence was present as women discussed the benefits of receiving weight gain feedback, whereas healthcare professionals did not describe any benefits.

*3d*. *Postnatal support*: There was complementarity between both populations feeling that obesity support beyond pregnancy was important (Table 6). However, there was dissonance between healthcare professionals’ description of the importance of postnatal support, and compliance with the pathways. Healthcare professionals described maternal obesity to have equal clinical and public health importance, and the need for collaborative work to support women beyond pregnancy. However, compliance with the pathways was poor for most public health oriented aspects of postnatal care, whereas it was acceptable for the clinical aspects.

*3e*. *Time and monetary costs for women*: The time commitment required from women for compliance with the pathways was a negative aspect of the pathways from women’s perspective (Table 6). This was a silent finding which healthcare professionals did not describe (they described their own time limitations in routine appointments but not the time commitment for women). However, healthcare professionals recognised the need for the pathways to address socio-economic status issues, and women reported the pathways to impose financial demands.

*3f*. *Information leaflets*: There was convergence, complementarity, dissonance and silence relating to the meta-theme of information leaflets (Table 6). This theme includes two leaflets intended for women and healthcare professionals. Women with a BMI>40kg/m^2^ were aware of the leaflets, and there was compliance with the provision of the leaflet to women in this BMI group. There was also complementarity between healthcare professionals’ and women’s views on the positive uses of leaflet, whereas only women expressed their limitations in changing behaviours. There was convergence between populations relating to the content of leaflets as both identified the need to include potential risks, practical tips and advice, and information on support services available. However, there were dissonant views on the priority of information to be included in leaflets: healthcare professionals viewed lifestyle information to be most important, whereas women had found the information on risks most useful. Healthcare professionals had limited awareness of the leaflet aimed to support their own practice, and their views on appropriate content for this resource largely reflect their views on training requirements.

## Discussion

Embarking on a mixed methods study such as this is important when neither qualitative nor quantitative methods alone would sufficiently answer a research question, and when mixing methods would yield a more complete analysis and robust study[48]. While the integration of mixed methods can be challenging, the use of survey methods and case note audit have provided breadth, while interviews have provided depth. Integration of these three studies has identified important aspects of agreement and disagreement relating to the implementation of maternal obesity care pathways. These areas of agreement and disagreement are likely to have an impact on compliance with care pathways, including both healthcare professionals’ compliance with delivering the pathways and women’s acceptance of the care required.

From the outset, researchers hope to find corroborating evidence when conducting mixed methods research[49,50], and there were multiple areas of agreement within the three meta-themes in this study. Healthcare professionals and women agreed that the care pathways were positive and beneficial in the *‘overall views’* meta-theme. The need for more consistent messages from healthcare professionals was identified within the *‘communication’* meta-theme by both populations, as was the influence of terminology on women’s response to weight-related discussions. Additionally, the clinical management (rather than public health management) aspects of the pathways were viewed positively with good compliance within the *‘content of the pathways’* meta-theme. The public health components were predominantly non-compliant, and both populations agreed that increased antenatal and postnatal weight management support was needed. This level of agreement can be used to make recommendations to address implementation limitations. For example, the development of training and support materials could promote more consistent compliance and communication among healthcare professionals, and existing services could be developed to ensure more equality in access to weight management support.

These examples of agreement show the benefits of finding corroborating evidence within mixed methods research. However, dissonance in mixed methods studies can be more complex to understand. Could dissonance reflect an inability to fully integrate data due to epistemological differences in qualitative and quantitative approaches? This is a philosophical argument against the use of mixed methods research[50–52], and literature recommends actively searching for discrepant data to identify ways of improving methodology for future studies to find more congruent results. However, the revelation of dissonant findings can also be useful if it adds to our depth of understanding[35,37]. The dissonance identified in this research revealed some of the most interesting, and potentially most important, findings. Active searching for dissonance during the integration stage identified differences between healthcare professionals’ and women’s priorities and perspectives, some of which were directly contradictory. Two key areas of dissonance which could potentially have a substantial impact on maternal obesity care relate to communication of weight and risk, and women’s engagement with weight management during pregnancy.

Healthcare professionals persistently identified the sensitivity and stigma of obesity to be a barrier to weight-related communication. This view is not uncommon, and was recently identified as a key barrier to healthcare professionals’ maternal obesity practice[33]. There is also evidence in the maternal obesity literature that obesity communication can be derogatory and upsetting for women. A systematic review identified women’s negative experiences of treatment from healthcare professionals as a central theme in several published studies; however, few studies included direct examples of negative treatment in the form of quotes[53]. The review identified that embarrassment and guilt were reported as feelings experienced by the women, especially at ultrasound appointments[53]. In our study, the only negative obesity-communication incident that was identified was also during an ultrasound appointment. In contrast to the perspectives of healthcare professionals who were caring for the women in our study, the majority of women viewed the communication approach from healthcare professionals to be positive and sensitive. For these women, it was the lack of adequate risk communication which was most likely to cause an emotive response, rather than issues of sensitivity. Further dissonance was apparent as healthcare professionals’ felt they were providing adequate explanations to women about obesity-related risk, and these aspects were generally compliant according to the women’s antenatal records. These differences in perspective are particularly important due to healthcare professionals’ primary focus on communication as a barrier to practice, whereas women felt that there needed to be a higher level of communication. Additionally, a lack of adequate risk communication could have an impact on the provision of safe care, which is a key component of the NHSLA CNST standards of care for maternity services[32].

The second important area of dissonance related to the different perspectives towards pregnant women’s engagement with weight management support. Healthcare professionals described women’s reluctance to engage with weight management advice and support when offered. A lack of engagement with weight management has been shown in other studies, with only 10% uptake of dietetic referrals among obese pregnancies[54], and 14.5% uptake among women eligible to participate in a weight management intervention[55]. Both of these studies identified a lack of promotion of weight management referrals by healthcare professionals to be a barrier to women’s engagement[54,55]. Additionally, a key barrier to the promotion of weight management referrals was healthcare professionals’ perception of the sensitivity of obesity discussions[55]. In contrast to the healthcare professionals’ perspectives in this study, the pregnant women considered weight management to be a priority, and placed importance on the fact that this support was missing from their care (when ineligible for the antenatal healthy lifestyle clinic). Additionally, there was a high level of engagement with the healthy lifestyle clinic (94%) and with the dietetic service within this clinic (91%) among eligible women. There was also overall satisfaction with the additional support provided through this clinic. Other research has identified that obese pregnant women are motivated for weight management support due to their priority of the health of their baby, even when they had refused support in the past[54]. Engagement with antenatal and postnatal weight management support has also been described to be mediated by parity, where multiparous women either had increased motivation, or different priorities driving their motivation[54,56].

In addition to active searching for dissonance, we also actively searched for silence. Silence can be considered similar to dissonance, in that it may be expected due to the strengths and limitations of different types of research approaches. However, *“surprise silences”* which are not expected can increase our understanding of the phenomenon under question[35]. Silence was observed in relation to two issues which women viewed as being important. When women received supportive weight-related feedback from healthcare professionals it had a positive impact on their self-esteem and motivation. Additionally, women felt that the time commitment required from them was a barrier to compliance with the pathways, particularly among those with other children or who worked. The absence of these issues from the healthcare professional respondent group again suggests a lack of shared priorities between participant populations. These differences in priorities are vital to shape service development to ensure healthcare professionals and women have a shared understanding. However, healthcare professionals did identify that improvements to the pathways should be women focussed, and involve women in their development. Despite the involvement of a multi-disciplinary group in the development of these pathways, there was an absence of patient and public involvement (PPI) apart from the review by the Maternity Services Liaison Committee. This evaluation has highlighted some important issues which may not have been apparent had there been more active PPI during the development of the pathways, rather than involvement at the review stage. The inclusion of women’s perspectives in the development of obesity-targeted services would facilitate increased women-centred care to encourage behaviour change, engagement, and patient satisfaction.

### Strengths and Limitations

A strength of this study is that it has explored the implementation of obesity care pathways from multiple perspectives within one healthcare setting, allowing for direct comparison. The integration of data has enabled a deeper understanding of some of the barriers and facilitators to the implementation of the pathways which may have been overlooked if we had used a sequential mixed methods model. For example, the direct comparison using integration methods identified important areas of disagreement in perspectives between pregnant women and the healthcare professionals who were looking after them. These are important findings that can be directly addressed in clinical practice to ensure that healthcare professionals and the women they care for have a shared understanding, agenda, and supportive care environment.

However, we must acknowledge that there are philosophical and practical limitations to mixed methods research with data integration. The mixed methods literature contains arguments that qualitative and quantitative approaches are underpinned by extensive epistemological differences, and there is a view that it is inappropriate to combine these approaches. Additionally, the practical difficulties and lack of published examples of data integration have led to the dominance of sequential models over simultaneous models. However, these arguments have been criticised as being unhelpful in progressing the methodology, and that a more pragmatic approach to mixed methods should be taken with published examples of the integration process[39]. We felt that a pragmatic approach was required for this study due to the potential benefits of exploring different population perspectives and compliance in one setting. We also felt that a direct comparison through data integration would add depth to our understanding, and we feel that this has been the case. Although there is an absence of published examples of data integration which is a limitation when developing your analytical plan, this evidence-base is increasing. A strength of our study is the use of a triangulation protocol, as recommended in the literature to attempt to manage the difficulties that can arise during data integration[35,37]. Data integration was a slow and challenging process, and deciding on the final method of integration to be included in the triangulation protocol took time to explore alternative methods, and teamwork absent of hierarchy to allow open and critical discussion of one another’s perspectives. However, once the triangulation protocol was developed it provided a model to help organise the data, and to be systematic while simultaneously having the freedom of interpretation.

A further potential limitation of this study is that three of the co-authors were involved in the multi-disciplinary group which developed the pathways of care (GS, HS, and NH), therefore potentially leading to author bias (i.e. wanting to find a favourable outcome to show effectiveness of the pathways). However, the purpose of the evaluation was to identify potential successes and failures relating to the implementation. Additionally, there were no financial or strategic incentives for intentionally producing favourable outcomes of the evaluation as the continued provision of the service was not linked to the evaluation. The remaining four authors (SD, SS, CDS and JR) were not involved in developing the pathways. Steps taken to avoid unintentional bias included the use of the project steering group (which included all seven co-authors) to develop the research design, data collection and analysis methods, and to monitor the research delivery. This included the development and refinement of the data collection tools (i.e. the interview schedule, questionnaire and audit criteria). The data analyses were also carried out by both NH and SD with agreement on the coding and interpretation of the data from each component study and during integration.

Finally, it was not possible to include the perspectives of dietitians within this study as at the time of study only one dietitian provided care for obese pregnant women and therefore we could not preserve anonymity.

## Conclusions

The development of care pathways for maternal obesity has provided healthcare professionals with structure and focus. This has facilitated the implementation of the clinical aspects of the pathways, particularly during the antenatal period. However, poor compliance with public health and postnatal aspects of the pathways suggests that further implementation work is required. Particular attention should be given to the differences in priorities between healthcare professionals and women at the developmental stage of care pathways, as well as the different perspectives on some of the approaches to implementation into routine practice. Developing shared perspectives between healthcare professionals and obese pregnant women could help facilitate more effective implementation of the pathway interventions which have poor compliance.

## Supporting Information

S1 Questionnaire Study 2 Healthcare Professional Questionnaire. (PDF)

S1 Table Care Pathway Interventions according to Booking BMI. (DOCX)

S2 Table Study 1 Interview Schedule. (DOCX)
