# Supplementary material for: An Evaluation of the Implementation of Maternal Obesity Pathways of Care: A Mixed Methods Study with Data Integration
Source: PLoS One. 2015 May 27;10(5):e0127122. doi: 10.1371/journal.pone.0127122 (PMC4446303; doi:10.1371/journal.pone.0127122)
Supplement: S1 Questionnaire — (PDF) [file pone.0127122.s001.pdf]

9. Health Professionals Information Leaflet continued (please tick one answer only):

	Strongly Agree	Agree	Neither Agree or Disagree	Disagree	Strongly Disagree
c) The information leaflet has not been useful to me	☐	☐	☐	☐	☐
d) The leaflet has helped me raise the issue of maternal obesity with women	☐	☐	☐	☐	☐
e) There is not enough information on the leaflet (if you agree with this statement please expand in Box 9)	☐	☐	☐	☐	☐
f) The leaflet is easy to follow	☐	☐	☐	☐	☐
g) The leaflet could be better (if you agree with this statement please expand in Box 9)	☐	☐	☐	☐	☐
h) The information in the leaflet is appropriate	☐	☐	☐	☐	☐

Box 9: What do you think a patient information leaflet about weight gain should include?

10. Additional points for discussion

a) Are there any aspects of the pathways particularly easy to deliver? Why?

b) Are there any aspects of the pathways particularly difficult to deliver? Why?

c) Are there any barriers to making the process work?

d) How could pathways be improved – with infinite finances? And in today's economic climate?

e) What words do you use when discussing BMI?

g) How do women respond when you talk about obesity?

g) Is there anything else you would like to add about the maternal BMI/obesity pathways?

Teesside University

South Tees Hospitals NHS
NHS Foundation Trust

Health Care Professional Staff Questionnaire

“An evaluation of antenatal, intrapartum and postnatal clinical pathways for obese pregnant women within South Tees NHS Trust maternity services, and partnership public health organisations”

Participant ID

Speciality (please tick one answer only):

☐ **Obstetrician** ☐ **Anesthetist** ☐ **Dietitian** ☐ **Health Improvement Specialist**

☐ **Registrar or SHO (Please circle type) Obstetrics / Anaesthetist**

☐ **Midwife (Please circle type) Community / Antenatal clinic / Delivery suite / Ward 17 / Maternity Assessment Unit**

We would like you to complete this questionnaire which should take no longer than 15 minutes.

Please answer the questions, and indicate how much you agree with each of the statements by ticking the options:

Strongly Agree	Agree	Neither Agree or Disagree	Disagree	Strongly Disagree
☐	☐	☐	☐	☐

You also have the option to expand on any of your responses for each question in the boxes provided.

You can also keep a reflective account of your experiences of the maternal BMI/Obesity pathways which can be used towards your continued professional development (CPD).

☐ I would like to keep a reflective account

☐ I do not want to keep a reflective account

Please return the questionnaire in the enclosed pre-paid envelope to:

Dr Nicola Heslehurst
Senior Lecturer in Research
Health and Social Care Institute
Parkside West Offices
Teesside University
Middlesbrough
TS1 3NN

If you have any difficulties completing the questionnaire, please contact:
Nicola Heslehurst on: **01642 342758**
email: **N.Heslehurst@tees.ac.uk**

Thank you

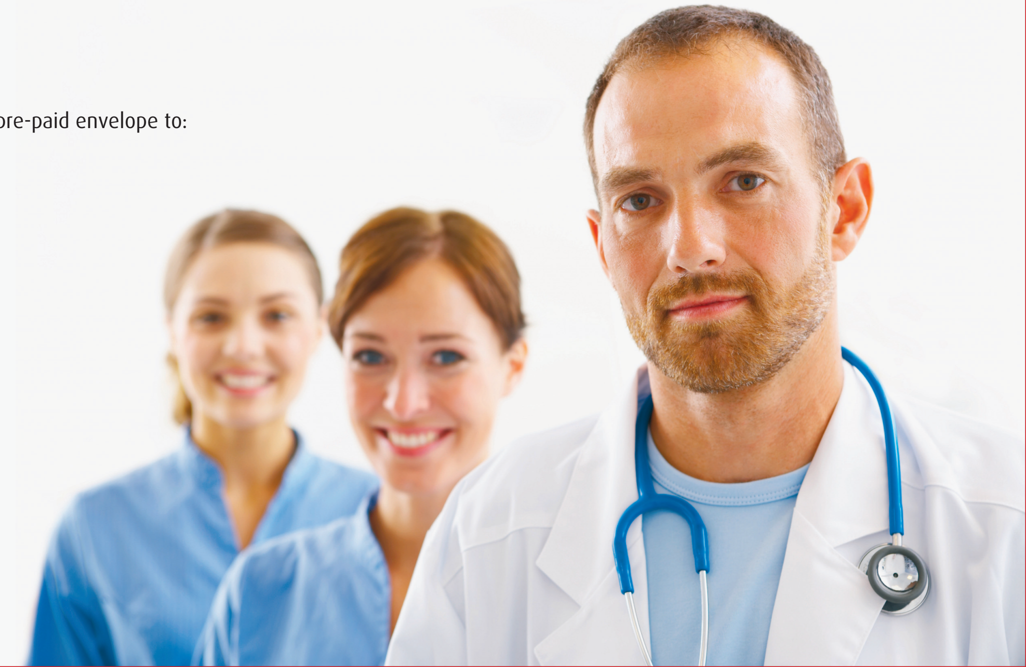

1. Are you aware that there are maternal BMI/obesity pathways? (please tick one answer only)

☐ Yes ☐ No

a. If you answered "Yes" then please continue to question 2.
b. If you answered "No" then what do you think should be included in these pathways?

You don't need to answer any further questions, thank you for your time.

2. How many pathways are there? ....

3. What are the BMI cut offs for the pathways? ....

4. Knowledge and understanding (please tick one answer only):

	Strongly Agree	Agree	Neither Agree or Disagree	Disagree	Strongly Disagree
a) I know why the maternal obesity pathways have been implemented	☐	☐	☐	☐	☐
b) I don't know why there are different pathways for different obesity groups	☐	☐	☐	☐	☐
c) I agree with the BMI cut offs used in the pathways	☐	☐	☐	☐	☐
d) Maternal obesity is an important clinical issue in pregnancy	☐	☐	☐	☐	☐
e) Maternal obesity is an important social issue in pregnancy	☐	☐	☐	☐	☐
f) Maternal obesity is a public health priority rather than an issue for maternity services	☐	☐	☐	☐	☐
g) I agree with the content of the pathways	☐	☐	☐	☐	☐
h) I would change some aspects of the pathways (if you agree with this statement please expand in Box 4)	☐	☐	☐	☐	☐

Box 4: Please expand on any of these issues if you would like to:

5. Confidence (please tick one answer only):

	Strongly Agree	Agree	Neither Agree or Disagree	Disagree	Strongly Disagree
a) I find it difficult to discuss BMI with obese pregnant women	☐	☐	☐	☐	☐
b) I am more confident in discussing the maternal obesity risk with patients since the implementation of the pathways	☐	☐	☐	☐	☐
c) I am less confident in discussing BMI status with patients since the implementation of the pathways	☐	☐	☐	☐	☐
d) I am more confident in giving weight gain advice in pregnancy to obese women since the implementation of the pathways	☐	☐	☐	☐	☐
e) I am confused about the weight gain advice I should be giving to women on the obesity pathways	☐	☐	☐	☐	☐
f) I don't feel qualified to discuss obesity with pregnant women	☐	☐	☐	☐	☐

Box 5: Please expand on any of these issues if you would like to:

6. Worthwhile (please tick one answer only):

	Strongly Agree	Agree	Neither Agree or Disagree	Disagree	Strongly Disagree
a) There has been an improvement in multi-disciplinary care since the implementation of the pathways	☐	☐	☐	☐	☐
b) I see the benefits in having maternal obesity pathways	☐	☐	☐	☐	☐
c) There are more disadvantages to the pathways than benefits (if you agree with this statement please expand in Box 6)	☐	☐	☐	☐	☐
d) The pathways are cost effective	☐	☐	☐	☐	☐
e) The facilities don't always allow compliance with pathways (if you agree with this statement please expand in Box 6)	☐	☐	☐	☐	☐
f) I think the pathways could be better (if you agree with this statement please expand in Box 6)	☐	☐	☐	☐	☐

Box 6: Please expand on any of these issues if you would like to:

7. Response from women (please tick one answer only):

	Strongly Agree	Agree	Neither Agree or Disagree	Disagree	Strongly Disagree
a) Discussing obesity upsets the patients	☐	☐	☐	☐	☐
b) I have experienced positive feedback from patients when I have discussed the obesity pathways	☐	☐	☐	☐	☐
c) Women are receptive to weight control advice in pregnancy	☐	☐	☐	☐	☐
d) Women don't understand why they are on the pathways	☐	☐	☐	☐	☐
e) Women don't accept that they are obese	☐	☐	☐	☐	☐
f) Women are compliant with the pathways during pregnancy	☐	☐	☐	☐	☐

Box 7: Please expand on any of these issues if you would like to:

8. Training Needs (please tick one answer only):

	Strongly Agree	Agree	Neither Agree or Disagree	Disagree	Strongly Disagree
a) I don't feel that I need any training for this issue	☐	☐	☐	☐	☐
b) I would benefit from some training around obesity in general	☐	☐	☐	☐	☐
c) I would benefit from training about the risks of maternal obesity	☐	☐	☐	☐	☐
d) I would benefit from some training about weight gain advice for obese women in pregnancy	☐	☐	☐	☐	☐
e) I would benefit from some training about the safety of dieting in pregnancy	☐	☐	☐	☐	☐
f) I would benefit from some training about the safety of exercise in pregnancy	☐	☐	☐	☐	☐
g) I would benefit from some training around sensitively discussing the issue of obesity with women	☐	☐	☐	☐	☐
h) I would benefit from some training but I'm not sure what in	☐	☐	☐	☐	☐

Box 8: Please expand on any of these issues if you would like to:

9. Health Professionals Information Leaflet: Raising issues of increased weight in pregnancy (please tick one answer only):

a) I have seen the staff information leaflet

☐ Yes ☐ No ☐ Not Sure

b) I have used the staff information leaflet

☐ Yes ☐ No (If no, please continue to Box 9)

Continued overleaf...
