# Supplementary material for: An Evaluation of the Implementation of Maternal Obesity Pathways of Care: A Mixed Methods Study with Data Integration
Source: PLoS One. 2015 May 27;10(5):e0127122. doi: 10.1371/journal.pone.0127122 (PMC4446303; doi:10.1371/journal.pone.0127122)
Supplement: S1 Table — (DOCX) [file pone.0127122.s002.docx]

**Supporting Information 1: Care pathway interventions according to booking BMI**

| **Contact with Healthcare Professional**  *Relevant pathway:*  Care Pathway 1:  Booking BMI 30-34.9kg/m^2^  Care Pathway 2:  Booking BMI 35-39.9 kg/m^2^  Care Pathway 3:  Booking BMI >40 kg/m^2^ | - **Intervention** |
| --- | --- |
| **Booking** | |
| *Care Pathways 1, 2 &3* | 1. Calculate and record BMI  2. Explain the implications of obesity  3. Discuss weight management in pregnancy  4. Provide information leaflet on healthy diet and physical activity in pregnancy  5. Give exercise advice  6. Offer folic acid 5mg  7. Offer vitamin D 10ug (Healthy Start Women’s vitamins or similar)  8. Book OGTT screening (if previous gestational diabetes screen at 16-18 weeks, and 28 weeks; for all other women screen at 24-28 weeks)  9. Offer random glucose test  10. Offer thyroid function test  11. Complete VTE Risk Assessment |
| *Plus for Care Pathway 2* | 12. Give advice regarding place of birth  13. Refer to High Dependency ANC **if** other co-morbidities  14. If no other co-morbidities discuss risks, weight management and exercise, complete alert card (BMI antenatal and delivery plan), MRSA screening, plan on-going antenatal care |
| *Plus for Care Pathway 3* | 15. Refer to High Dependency ANC **if** co-morbidities present; 16. Refer to healthy lifestyle clinic if **no** co-morbidities present |
| **High Dependency Antenatal Clinic (ANC)** | |
| *Care Pathways 2&3* | 1. Discuss risks of raised BMI in pregnancy  2. Discuss weight management and exercise  3. Complete alert card in hand held notes for BMI, antenatal plan, delivery plan  4. Undertake MRSA screening as per protocol  5. Plan for on-going antenatal care  6. Book anaesthetic review **if** co-morbidities |
| *Plus for Care Pathway 3* | 7. Book anaesthetic review **for all** |
| **On-going Antenatal Care** | |
| *Care Pathways 1, 2 &3* | 1. Use appropriate BP cuff, document size of cuff used. Refer to hypertension guideline if BP abnormal  2. Offer continued encouragement and support regarding diet and activity  3. Provide information on infant feeding to provide informed choice  4. Thromboprophylaxis assessment |
| **20- 30 weeks** | |
| *Care Pathway 2* | 1. Anaesthetic referral **if** co-morbidities |
| *Care Pathway 3* | 2. Anaesthetic referral BMI 40-45; anaesthetic review BMI >45 |
| **28 weeks** | |
| *Care Pathways 1, 2 &3* | 1. Weigh and calculate weight gain |
| *Plus for Care Pathway 3* | 2. Individual manual handling risk assessment – advice and action plan |
| **32 weeks** | |
| *Care Pathway 3* | 1. USS for growth and fetal wellbeing  2. Weigh and calculate weight gain |
| **34-36 weeks** | |
| *Care Pathways 1, 2 & 3* | 1. Weigh and calculate weight gain  2. Ensure infant feeding check list has been fully completed |
| *Plus for Care Pathways 2 & 3* | 3. Give advice regarding place of birth  4. Positions in labour discussed to support normal birth |
| *Plus for Care Pathway 3* | 5. USS for growth and fetal well being |
| **Antenatal Admission** | |
| *Care Pathways 1, 2 & 3* | 1. Antenatal thromboprophylaxis assessment if admitted for >24hrs |
| *Plus for Care Pathway 3* | 2. Preparation for Labour: ensure appropriate equipment available if booked for IOL or LSCS |
| **Labour** | |
| *Care Pathways 1, 2 &3* | 1. Active management of 3^rd^ stage of labour recommended and documented in notes  2. IV cannula if antenatal thromboprophylaxis administered, APH or previous PPH  3. *Care pathway 1 only:* if no other risk factors - suitable for low dependency labour ward |
| *Plus for Care Pathways 2 & 3* | 4. Not suitable for low dependency labour ward  5. If no other risk factors - can follow low dependency protocols  6. Inform duty anaesthetist need for anaesthetic review  7. Ranitidine as per protocol  8. Pressure care guidelines  9. TED stocking to be worn throughout labour  10. Decision for caesarean to be at consultant level, consider need for consultant to be present depending on other risk factors  11. Consultant anaesthetist to be informed if surgery is anticipated  12. Theatre teams to be informed if woman weighs over 120kg in labour  13. If caesarean section undertaken >2cm of subcutaneous fat to have suturing of subcutaneous space |
| *Plus for Care Pathway 3* | 14. Active management of 3^rd^ stage of labour recommended and documented in notes  15. IV cannula in labour  16. If caesarean section undertaken prophylactic antibiotics required at time of surgery  17: FBC, group and save  18. Continuous CTG monitoring, may need FSE  19. Inform duty anaesthetist need for anaesthetic review  20. Inform consultant obstetrician and early involvement sought as required  21. Decision for caesarean to be at consultant level, ideally consultant should be present for caesarean |
| **Postnatal** | |
| *Care Pathways 1, 2 & 3* | 1. Postpartum thromboprophylaxis assessment, encourage early and regular ambulation  2. Breastfeeding support  3. Contraceptive advice  4. Provide information on commencing folic acid 5mgs 1 month before stopping contraception  5. Provide healthy lifestyle advice and signpost to postnatal services |
| *Plus for Care Pathways 2 & 3* | 6. On-going support from community based dietetics services |
| *Plus for Care Pathway 3* | 7. Strict attention to wound and perineal care |
