# Supplementary material for: An Evaluation of the Implementation of Maternal Obesity Pathways of Care: A Mixed Methods Study with Data Integration
Source: PLoS One. 2015 May 27;10(5):e0127122. doi: 10.1371/journal.pone.0127122 (PMC4446303; doi:10.1371/journal.pone.0127122)
Supplement: S2 Table — (DOCX) [file pone.0127122.s003.docx]

**Supporting Information 2: Study 1 Interview Schedule**

| **Question/Topic** | **Additional prompts if necessary** |
| --- | --- |
| Can you remember if you had your height and weight measured at your booking appointment? | - - What do you understand about BMI?   - What was explained to you about your BMI? |
| Have the BMI pathways been explained to you? | - - What do you understand about the pathways?   - How was the pathway explained to you? |
| Do you have a sheet like this in your notes? (Show proforma) | - - What was explained to you about it?   - What do you understand about why it is there? |
| How do you feel about being on the pathways? | - - Is there anything particularly good about it?   - Is there anything particularly bad about it? |
| What support with your BMI do you think you would like in pregnancy? | - - What other services would you find useful?   - Have you been offered a referral to a dietitian or anything like that?   - Would you/have you seen a dietitian? Why? |
| What advice have you been given about weight gain? | - What were you told about weight gain? |
| Did you see or get a copy of this leaflet (show them the local patient information leaflet on healthy lifestyles)? | - - If they have seen the leaflet: What did you think of it? How clear was the information?   - If they have not seen the leaflet (read through the leaflet together and give them time to read through on their own): What are your first impressions of the leaflet? How clear is the information?   - Is there anything particularly good or bad about it?   - Is there anything that you think should be on there that isn’t? |
